# Avian leukosis virus subgroup J evades innate immunity by activating miR-155 to dually target TRAF3 and STAT1

**DOI:** 10.1371/journal.ppat.1013552

**Published:** 2025-10-09

**Authors:** Xinyue Zhang, Defang Zhou, Jing Zhou, Xiaoyang Liu, Longying Ding, Tianxing Yan, Mengyuan Shi, Shuhai He, Ziqiang Cheng

**Affiliations:** 1 College of Veterinary Medicine, Shandong Agricultural University, Tai’an City, Shandong Province, China; 2 College of Animal Science and Technology, Xinyang Agriculture and Forestry University, Xinyang City, Henan Province, China; University of Illinois at Chicago College of Medicine, UNITED STATES OF AMERICA

## Abstract

Avian leukosis virus subgroup J (ALV-J) causes immunosuppression and myelocytomas in poultry. While ALV-J evades innate immunity to sustain infection, the mechanisms remain unclear. Here, we show that ALV-J upregulates microRNA-155 (miR-155) to suppress type I interferon (IFN-I) responses, facilitating viral replication. Mechanistically, the ALV-J p27 protein reduced DEAD-box helicase 3X (DDX3X) promoter activity, repressing its expression and promoting MIR155 host gene (MIR155HG) processing into mature miR-155. miR-155 directly targets the tumor necrosis factor receptor-associated factor 3 (TRAF3) and the signal transducer and activator of transcription 1 (STAT1), both of which are key regulators of IFN-I response. Silencing TRAF3 or STAT1 rescues ALV-J replication suppressed by miR-155 inhibition. These findings reveal a novel miRNA-mediated innate immune evasion strategy employed by ALV-J, enhancing our understanding of retroviral pathogenesis.

## Author summary

Avian leukosis virus subgroup J (ALV-J) causes severe economic losses in poultry by suppressing immunity and inducing tumors. This study uncovers how ALV-J hijacks a host microRNA, miR-155, to evade antiviral defenses. We found that ALV-J p27 protein silences DEAD-box helicase 3X (DDX3X), a cellular protein involved in RNA processing, leading to increased miR-155 levels. miR-155 then blocks the activity of two critical immune proteins, TRAF3 and STAT1, which are essential for triggering antiviral responses. By disrupting these proteins, ALV-J reduces interferon production and signaling, allowing the virus to replicate unchecked. This discovery highlights a sophisticated mechanism by which viruses manipulate host molecules to persist and cause disease.

## Introduction

Avian leukosis virus subgroup J (ALV-J), an oncogenic retrovirus with rapid evolutionary dynamics, induces severe immunosuppression and neoplastic pathologies including myelocytomas and hemangiomas in poultry [1,2]. These conditions result in growth retardation, opportunistic infections, and reduced productivity, causing substantial economic losses in commercial flocks [3–5]. Both horizontal and vertical transmission routes have been documented, with vertical transmission often resulting in more severe pathogenicity [4,6]. Infections during embryonic development or early life frequently progress to chronic viral persistence, creating a permissive microenvironment for myeloid leukemogenesis and other tumors through sustained immunosuppression and oncogenic transformation [7,8]. Central to this pathogenesis is the virus-host interplay between immune surveillance mechanisms and viral immune evasion strategies [9,10]. Evading the host immune response is essential for ALV-J to establish infection, achieve lifelong persistence in the host successfully, and enable tumorigenesis. However, the precise mechanism remains poorly characterized.

ALV-J deploys multifaceted immune subversion tactics to sustain persistent infection despite host antiviral defenses. Its myeloid cell tropism results in disrupting hematopoietic differentiation, triggering apoptosis while affecting cytokine production, inflammatory responses, and antigen presentation, collectively fostering immunosuppressive niches [11–14]. Embryonic exposure induces antigen-specific immune tolerance, evidenced by absent neutralizing antibodies despite prolonged high-titer viremia [8,15]. Additionally, the high mutation rate of reverse transcriptase, genomic recombination, and variability in cell-mediated immune responses contribute to a diverse population of ALV-J variants that can persist in the host and avoid immune clearance [16–18]. Notably, as an obligate intracellular parasite, ALV-J hijacks host factors, including microRNAs (miRNAs), to combat antiviral defenses and facilitate virus replication [19–22]. However, the mechanism underlying ALV-J-induced immune evasion is not fully understood, necessitating further research to elucidate these processes.

miRNAs are evolutionarily conserved non-coding RNAs that regulate gene expression through complementing to sequences in the 3′ untranslated region (UTR) of messenger RNAs (mRNAs), leading to mRNA decay and translational repression [23,24]. As potent post-transcriptional regulators of gene expression, miRNAs actively act in innate and adaptive immunity and become appealing candidates for viral immune-evasion mechanisms [25–27]. Indeed, several oncogenic viruses hijack host miRNA pathways to facilitate viral persistence and malignant transformation [28,29]. In a similar vein, ALV-J has acquired a solid ability to manipulate cellular miRNA pathways to generate favorable conditions for the ALV-J lifecycle, tumorigenesis, and immunosuppression during coevolution with its host. For instance, gga-miR-200b-3p facilitates ALV-J replication by targeting the host protein dual-specificity phosphatase 1 (DUSP1). ALV-J activates tumor-associated miRNAs, contributing to the carcinogenic processes [30,31]. Furthermore, ALV-J inhibits the interferon (IFN) signaling pathway through miRNA activation. The expression of miR-34b-5p and miR-23b is significantly upregulated in ALV-J-infected chicken spleens, and the expression of miR-34b-5p targeting the melanoma differentiation-associated protein 5 (MDA5) signaling pathway promotes ALV-J replication levels [21]. miR-23b directly targeted the interferon regulatory factor 1 (IRF1), thereby decreasing IFN-β expression [22]. Therefore, identifying the miRNA fingerprints of ALV-J infection and elucidating their various roles in pathogenesis is particularly interesting.

## Results

### ALV-J suppresses the IFN-I response and enhances miR-155 expression

**Fig 1:**
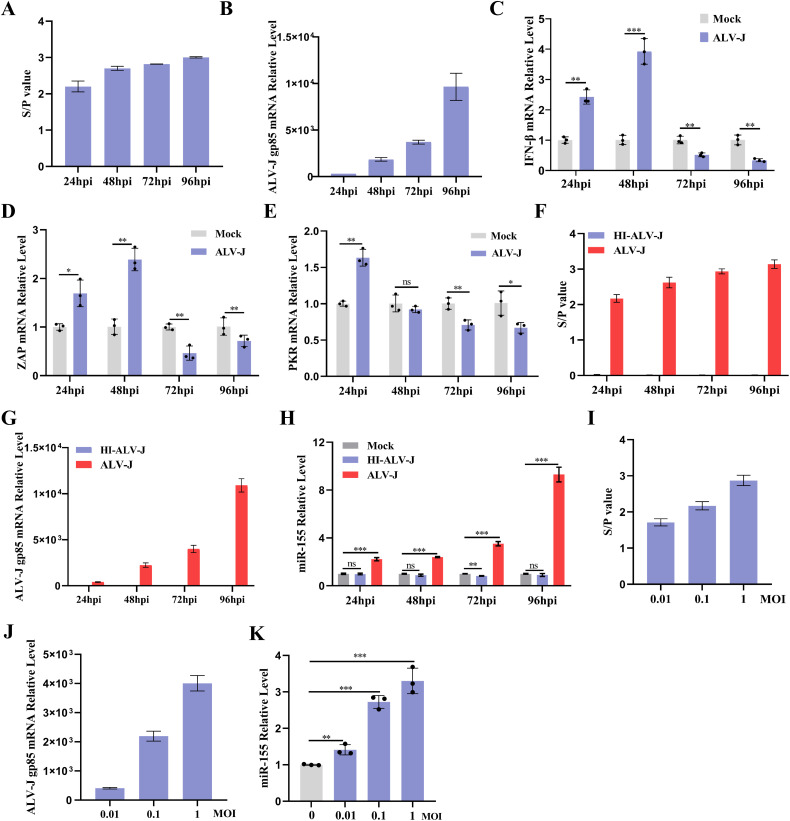
ALV-J suppresses IFN-I response and activates miR-155 expression. **(A-E)** ALV-J suppresses the induction of IFN-β and ISGs during the late phase of infection. CEFs were infected with ALV-J at an MOI of 1 for the indicated times. (A) ELISA detection of p27 protein levels in cell supernatants. The transcription levels of ALV-J gp85 (B), IFN-β (C), ZAP (D), and PKR (E) were measured using qRT-PCR and normalized to GAPDH. **(F-K)** ALV-J enhances miR-155 expression. (F-H) CEFs were infected with ALV-J at an MOI of 1 for the indicated times. (I-K) CEFs were infected with ALV-J at the specific MOI for 72 **h.** (F and I) The p27 proteins in the culture supernatant were measured by ELISA. (G and J) The levels of ALV-J gp85 mRNA were evaluated using qRT-PCR. (H and K) The level of miR-155 was measured by qRT-PCR and normalized to U6. *, P < 0.05. **, P < 0.01. ***, P < 0.001. ns, P > 0.05, no significant difference.

To clarify the impact of ALV-J load on the production of IFN-β and IFN-stimulated genes (ISGs) in infected cells, CEFs were infected with ALV-J. ALV-J infection was confirmed by ELISA detection of p27 protein levels in cell supernatants and quantitation reverse transcription PCR (qRT-PCR) detection of ALV-J gp85 mRNA levels (Fig 1A and 1B). The mRNA expression levels of IFN-β and ISGs were analyzed using qRT-PCR. As shown in Fig 1C, IFN-β induction peaked at 24–48 hpi, followed by significant diminution at 72–96 hpi. Concurrently, ALV-J infection induced marked suppression of ISGs Zinc-finger antiviral protein (ZAP) and protein kinase R (PKR) transcription during late infection phases (Fig 1D and 1E). To identify potential factors involved in IFN response induced by ALV-J, we analyzed the expression profile of miRNAs. Previous transcriptomic analyses from our group identified significant upregulation of miR-155 in ALV-J-infected CEFs [32]. CEFs possess a complete miRNA processing machinery (S1 Fig). To determine the relationship between ALV-J infection and miR-155 activation, we assessed miR-155 expression in CEFs infected with ALV-J (multiplicity of infection [MOI]=1), inoculated with heat-inactivated ALV-J (HI-ALV-J; 70°C for 60 min), or Mock treatment in a time-course assay. Infection status in these experiments was validated by p27 ELISA and gp85 qRT-PCR (Fig 1F and 1G). The results showed that live virus induced miR-155 expression, while HI-ALV-J did not affect the expression levels of miR-155 (Fig 1H), suggesting that the increased miR-155 was dependent on ALV-J replication. Moreover, the expression of miR-155 was enhanced by ALV-J infection in a dose-dependent manner (Fig 1I-1K). These results indicated that ALV-J infection inhibits the IFN response and activates miR-155 expression.

### ALV-J represses DDX3X and promotes the accumulation of mature miR-155

**Fig 2:**
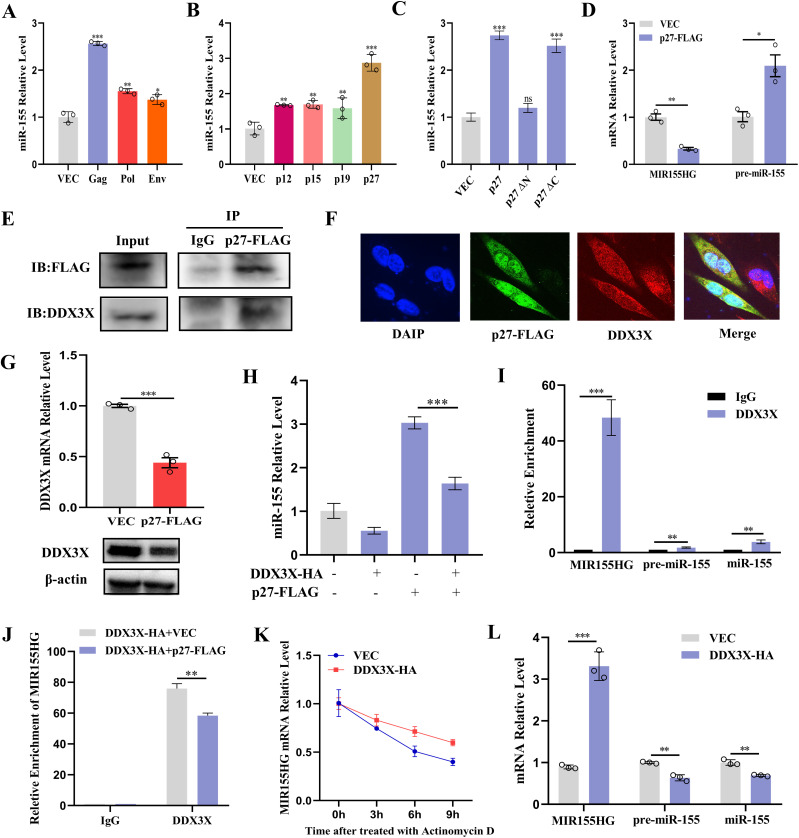
ALV-J p27 represses DDX3X and activates miR-155 expression. **(A-C)** ALV-J p27 protein N-terminal domain activates miR-155 expression. CEFs were transfected with the indicated plasmids for 48 h. The miR-155 levels were measured using qRT-PCR. **(D)** CEFs were transfected with p27 plasmid for 48 h. The MIR155HG and pre-miR-155 levels were measured using qRT-PCR. **(E and F)** ALV-J p27 protein interacts with DDX3X. (E) Interaction of endogenous DDX3X with ALV-J p27. CEFs were transfected with p27-FLAG for 48 h before coimmunoprecipitation and immunoblot analysis with the indicated antibodies. Mouse IgG antibodies were utilized as a control. (F) Confocal microscopy analysis indicating co-localization of exogenous p27 and DDX3X in the cytoplasm and nucleus. **(G)** ALV-J p27 protein represses endogenous DDX3X levels. CEFs were transfected with the p27-FLAG plasmids for 48 h. The DDX3X mRNA and protein levels were determined using qRT-PCR and Western blotting. **(H)** Overexpression of DDX3X inhibits p27-mediated miR-155 activation. CEFs were transfected with p27-FLAG plasmid, DDX3X-HA plasmid, or both for 48 h, and then the miR-155 levels were measured using qRT-PCR. **(I and J)** RNA immunoprecipitation of DDX3X. (I) CEFs were transfected with the DDX3X-HA plasmid for 48h, and immunoprecipitations were performed with anti-DDX3X antibodies. RNA was extracted from immunoprecipitates and checked for the levels of MIR155HG, pre-miR-155, and miR-155 using qRT-PCR. Rabbit IgG antibodies were used as a control. (**J**) CEFs were co-transfected with p27-FLAG or corresponding control and DDX3X-HA plasmid, and immunoprecipitations were performed with anti-HA antibodies. RNA was extracted from immunoprecipitates and checked for the levels of MIR155HG using qRT-PCR. Rabbit IgG antibodies were used as a control. **(K)** MIR155HG stability assay. CEFs were transfected with DDX3X-HA plasmid or corresponding control for 24h, then treated with the transcription inhibitor actinomycin D (5 μg/mL). MIR155HG was quantified by qRT-PCR at 0, 3, 6, and 9 h. **(L)** CEFs were transfected with DDX3X-HA plasmid or corresponding control for 12 h, and then infected with ALV-J for 36 h, qRT-PCR analysis of the level of miR-155, pre-miR-155, and MIR155HG. *, P < 0.05. **, P < 0.01. ***, P < 0.001. ns, P > 0.05, no significant difference.

To determine the viral component responsible for miR-155 activation, we transfected plasmids encoding ALV-J structural proteins (Gag, Pol, and Env) into chicken CEFs and quantified miR-155 expression via qRT-PCR. As illustrated in Fig 2A, all three proteins significantly upregulated miR-155, with Gag demonstrating superior induction capacity. As Gag is processed into p19, p27, p12, and p15 subunits, we performed subunit-specific analyses and identified p27 as the dominant driver of miR-155 activation (Fig 2B). Due to p27 containing N- and C-terminal domains (akin to HIV p24), we generated a panel of truncation variants (S2 Fig) and found the miR-155 inducing activity to reside in the N-terminal domain: constructs lacking the N-terminus lost activity, whereas those retaining it preserved induction (Fig 2C). We next investigated whether p27 regulates miR-155 transcriptionally or post-transcriptionally. Measurement of MIR155 host gene (MIR155HG) and pre-miR-155 revealed that p27 overexpression reduced MIR155HG transcript levels but increased both pre-miR-155 and mature miR-155, indicating post-transcriptional enhancement of miR-155 biogenesis (Fig 2D).

To elucidate the molecular mechanism, we employed affinity purification and mass spectrometry (AP-MS) to identify p27-interacting partners in CEFs. MS analyses identified proteins specifically associated with p27 (S1 Table). Among the candidates, DEAD-box helicase 3X (DDX3X), an RNA helicase critical for miRNA biogenesis, is a key interactor. We then tested whether any interaction between DDX3X and p27 occurred. Co-immunoprecipitation (Co-IP) confirmed the physical interaction between FLAG-tagged p27 and endogenous DDX3X (Fig 2E). To determine the subcellular localization of the p27-DDX3X interaction, we performed nuclear-cytoplasmic fractionation followed by Co-IP. The results showed that p27 and DDX3X formed complexes in both the cytoplasmic and nuclear fractions, indicating that their interaction occurs in both compartments (S3 Fig). Consistently, immunofluorescence staining further demonstrated colocalization of p27 and DDX3X in the cytoplasm as well as in the nucleus, corroborating the Co-IP findings (Fig 2F). Mapping experiments localized the interaction to the N-terminal domain of p27 (S4 Fig). Functional studies showed p27-mediated suppression of DDX3X expression at both transcriptional and translational levels (Fig 2G). This suppression was mediated specifically through the N-terminal domain of p27, which significantly reduced DDX3X promoter activity without affecting mRNA or protein stability (S5 Fig).

Notably, overexpression of DDX3X effectively reversed p27-induced miR-155 activation (S6A and 2H Figs). To validate whether other miRNAs are affected by p27-DDX3X-mediated regulation, we also examined other miRNAs upregulated in the sequencing data, including miR-146a-5p, let-7b, and miR-221. qRT-PCR analysis showed that while p27 modestly induces these miRNAs, overexpression of DDX3X most significantly reversed the activation of miR-155 induced by p27 (S7 and 2H Figs). These results suggest that while p27 influences multiple miRNAs, miR-155 is the principal target of p27-DDX3X-mediated regulation. To determine whether DDX3X was directly involved in the miR-155 biogenesis, we performed RNA immunoprecipitation (RIP). RIP analysis with DDX3X revealed the most significant enrichment with MIR155HG (Fig 2I), and importantly p27 overexpression markedly decreased DDX3X association with MIR155HG (Fig 2J). Since members of the DEAD-box protein family have been shown to participate in primary miRNA processing and mRNA decay, we investigated the potential effect of DDX3X on the regulation of MIR155HG by an RNA stability assay. Actinomycin-D chase experiments revealed that DDX3X increases MIR155HG transcript stability, while interference with DDX3X resulted in reduced transcript stability (Figs 2K, S6B, and S8A). Consistent with these observations, during ALV-J infection, DDX3X overexpression increased MIR155HG levels while lowering pre- and mature miR-155, whereas DDX3X depletion produced the opposite pattern (Figs 2L and S8B). These results suggest that p27 represses DDX3X and interferes with DDX3X-MIR155HG interaction, thereby reducing MIR155HG stability and promoting accumulation of mature miR-155.

### miR-155 suppresses IFN-I response to promote ALV-J replication

**Fig 3:**
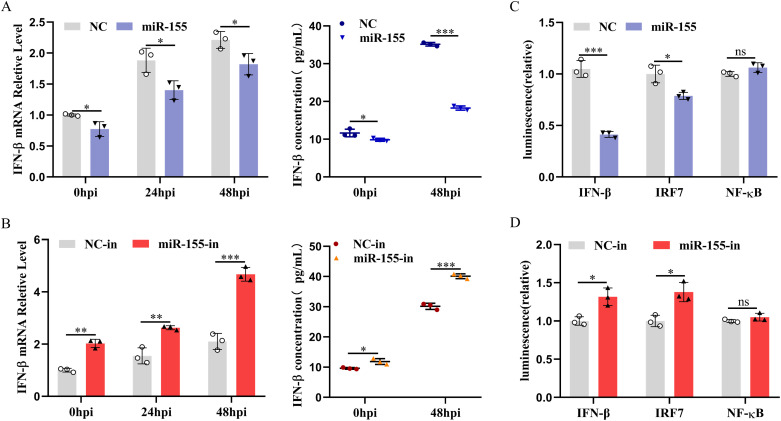
miR-155 reduces IFN-β production. **(A and B)** miR-155 suppresses IFN-β production. CEFs were transfected with 30 nM miR-155 mimics, 60 nM miR-155 inhibitor (miR-155-in), or their corresponding control oligonucleotides for 12 h, and then the cells were infected with ALV-J at an MOI of 1 for the indicated time. Then, the transcription levels of IFN-β were measured using qRT-PCR, and IFN-β in supernatants was measured by ELISA. **(C and D)** miR-155 inhibits IFN-β promoter activity by inhibiting IRF7 activation. IRF7, NF-κB, or IFN-β promoter along with pRL-TK were co-transfected with 30 nM miR-155 mimics, 60 nM miR-155-in or their corresponding control oligonucleotides for 36 h, followed by incubation with poly (I:C) (5 μg/mL) for 12 h. Then, cells were harvested for the luciferase assay. *, P < 0.05. **, P < 0.01. ***, P < 0.001. ns, P > 0.05, no significant difference.

To gain insight into the role of miR-155 in regulating innate immune responses during ALV-J infection, we investigated whether miR-155 alters interferon-I (IFN-I) production and signaling. We found that miR-155 overexpression inhibited ALV-J-triggered IFN-β production at both the mRNA and protein levels (Fig 3A). In contrast, inhibition of miR-155 promoted ALV-J-triggered IFN-β production (Fig 3B). Since chickens lack IFN regulatory factor 3 (IRF3), the enhancement of IFN depends on the binding of IRF7 and NF-κB transcription factors to their respective regulatory domains in the promoter region of the IFN gene [33,34]. To determine whether miR-155 suppressed IFN-β production by regulating these transcriptional factors, we used a dual-luciferase reporter assay to monitor the activation of NF-κB, IRF7, and IFN-β promoter after poly(I:C) stimulation. Consistent with the inhibitory effect of miR-155 on IFN-I production, overexpression of miR-155 attenuated IFN-promoter activity substantially (Fig 3C). A similar activation of IFN-promoter by miR-155 inhibitor was also observed (Fig 3D). Further results revealed that miR-155 reduced IRF7 reporter activity, but did not significantly affect NF-κB reporter activity (Fig 3C and 3D). These results suggested that miR-155 suppressed IFN-β production by inhibiting IRF7 activation without affecting NF-κB.

**Fig 4:**
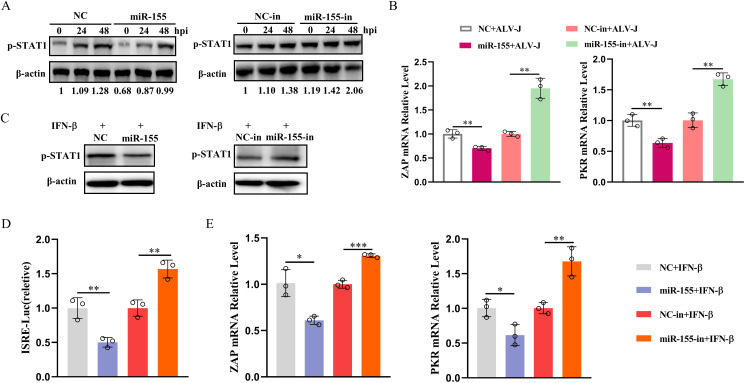
miR-155 impairs the IFN-I signaling. **(A and B)** miR-155 attenuates the IFN-I signaling during ALV-J infection. CEFs were transfected with 30 nM miR-155 mimics, 60 nM miR-155-in, or their corresponding control oligonucleotides for 12 h, and then infected with ALV-J at an MOI of 1 for the indicated times. The expression levels of phosphorylated STAT1 were detected by Western blotting (A). The transcription levels of ISGs were measured using qRT-PCR (B). **(C-E)** miR-155 impairs antiviral responsiveness of IFN-β. DF-1 cells were transfected with 30 nM miR-155 mimics, 60 nM miR-155-in, or their corresponding control oligonucleotides. After 36 h, cells were incubated with recombinant chicken IFN-β (200 ng/mL) for 12 h. Phosphorylated STAT1 levels were detected by Western blotting (C), and the transcription levels of ISGs were measured by qRT-PCR (E). (D) The ISRE reporter vectors along with pRL-TK were co-transfected with 30 nM miR-155 mimics, 60 nM miR-155-in, or their corresponding control oligonucleotides for 36 h. Cells were incubated with recombinant chicken IFN-β (200 ng/mL) for 12 h before harvesting for the luciferase assay. *, P < 0.05. **, P < 0.01. ***, P < 0.001.

Next, we investigated whether miR-155 impacts IFN-β-induced signaling. IFN-I activates the JAK-STAT signaling pathway to induce rapid and robust transcriptional induction of ISGs, which inhibit viral infection. Transcription of ISGs is dependent on signal transducer and activator of transcription 1 (STAT1) phosphorylation and the activation of IFN-sensitive response elements (ISRE) [35]. In ALV-J-infected CEFs, miR-155 overexpression inhibited STAT1 phosphorylation and the expression of ISGs mRNA, whereas suppression of miR-155 exerted the opposite effect (Fig 4A and 4B). To confirm these findings, we treated miR-155-transfected DF-1 cells with IFN-β to directly initiate IFN-I signaling and further assessed STAT1 phosphorylation, ISRE promoter activity, and ISG expression. As expected, overexpression of miR-155 attenuated IFN-β-induced STAT1 phosphorylation (Fig 4C), ISRE promoter activity (Fig 4D), and ISG expression (Fig 4E), while miR-155 inhibition exerted the opposite effects. The results indicated that miR-155, by reducing STAT1 phosphorylation, ISRE promoter activity, and downstream ISG responses, mediates the interference with IFN-I signaling.

**Fig 5:**
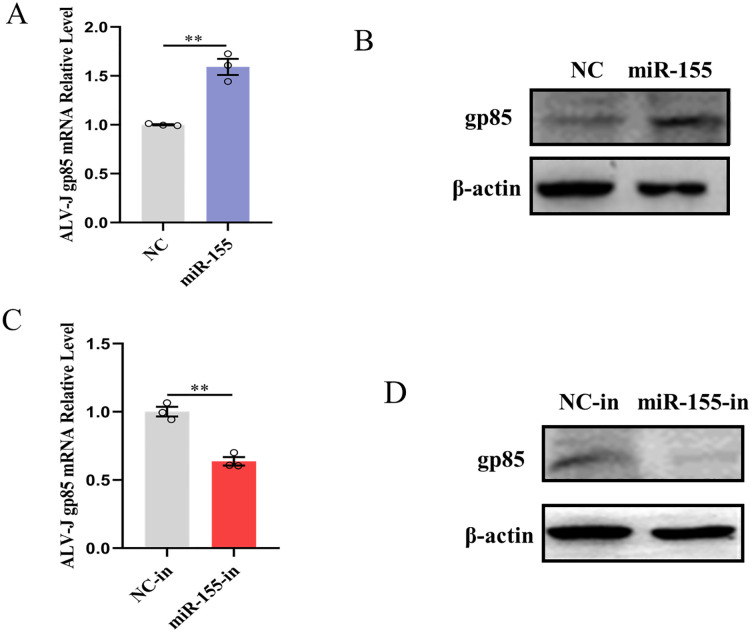
miR-155 facilitates ALV-J replication. **(A-D)** miR-155 promotes ALV-J replication. CEFs were transfected with 30 nM miR-155 mimics, 60 nM miR-155-in, or the corresponding control oligonucleotides for 12 h. Subsequently, cells were infected with ALV-J at an MOI of 1. At 36 h post-infection, the levels of ALV-J gp85 mRNA and protein were respectively evaluated by qRT-PCR and Western blotting. **, P < 0.01.

To evaluate the possible effect of miR-155 on ALV-J replication, miRNA gain-of-function and loss-of-function experiments were conducted. CEFs were transfected with miR-155 mimics or miR-155 inhibitor and then infected with ALV-J at an MOI of 1. Replication of the virus was monitored by measuring intracellular ALV-J gp85 mRNA and protein levels. The results indicated that miR-155 overexpression significantly increased the abundance of ALV-J gp85 mRNA and protein (Fig 5A and 5B). Moreover, when the miR-155 inhibitor was applied, ALV-J gp85 mRNA and protein abundance decreased significantly (Fig 5C and 5D). These findings indicated that miR-155 enhanced ALV-J replication.

These findings suggested that ALV-J-induced miR-155 restricts IFN-β production and signaling to surmount host innate antiviral immune responses, thereby facilitating its replication.

### miR-155 targets TRAF3 and STAT1

**Fig 6:**
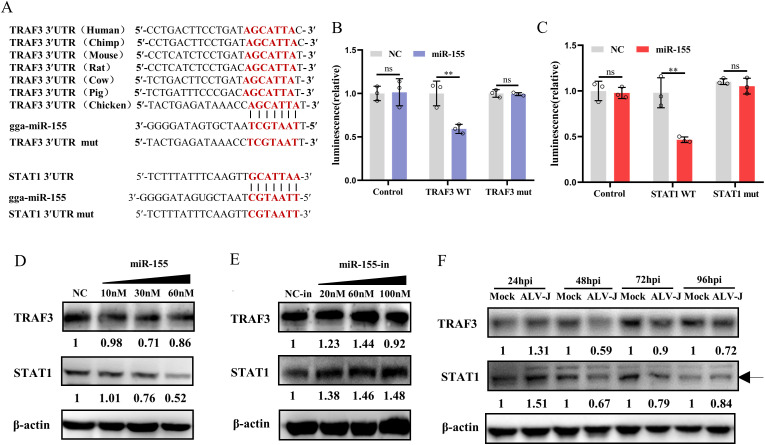
miR-155 directly targets the 3′UTR of TRAF3 and STAT1. **(A)** The predicted target sites of miR-155 in TRAF3 and STAT1 3′UTRs, as well as the mutant forms with the potential miR-155 binding sites mutated. **(B and C)** miR-155 targets the 3′UTR of TRAF3 and STAT1. 293T cells were co-transfected with a luciferase reporter vector containing the WT or mutant 3′UTR of TRAF3 or STAT1 (100 ng) and 30 nM miR-155 mimics or NC for 48 h, and then harvested for luciferase assay. **(D and E)** miR-155 inhibits protein levels of endogenous TRAF3 and STAT1. CEFs were transfected with miR-155 mimics, 60 nM miR-155-in, or the corresponding control oligonucleotides. Protein levels of endogenous TRAF3 and STAT1 were analyzed by Western blotting. **(F)** TRAF3 and STAT1 expression is decreased during the late phase of ALV-J infection. CEFs were infected with ALV-J at an MOI of 1 for the indicated times. The levels of TRAF3 and STAT1 protein were detected by Western blotting. **, P < 0.01. ns, P > 0.05, no significant difference.

To investigate the primary targets of miR-155 involved in modulating IFN-I response, computational analysis was performed using three different miRNA binding-site prediction programs: TargetScan, miRDB, and miRanda. This analysis revealed miR-155 binding sites within the 3′UTR regions of tumor necrosis factor receptor-associated factor 3 (TRAF3) and STAT1 (Fig 6A). To validate these predictions experimentally, we constructed wild-type (WT) and mutated reporter plasmids. Transfection of the miR-155 mimic significantly reduced the luciferase gene expression carried by the WT TRAF3 and STAT1 reporter construct but not that carried by the corresponding scrambled controls. Mutations in the miR-155 binding sites of TRAF3 and STAT1 led to significant recoveries of luciferase levels (Fig 6B and 6C). The results of reporter assays indicated a direct interaction of miR-155 with predicted target sites in the 3′UTR of TRAF3 and STAT1 mRNA. We further assessed endogenous TRAF3 and STAT1 protein regulation by miR-155 in CEFs. Western blotting showed that miR-155 mimic transfection reduced TRAF3 and STAT1 levels, whereas miR-155 inhibitor increased their expression (Fig 6D and 6E). Because miR-155 is upregulated by ALV-J, and overexpression of miR-155 impairs TRAF3 and STAT1 expression, we speculated that TRAF3 and STAT1 expression might be downregulated during ALV-J infection. To test this, we examined the expression kinetics of TRAF3 and STAT1 during ALV-J infection. Western blotting results indicated that TRAF3 and STAT1 protein levels accumulated dramatically within 24 h after the ALV-J attack but decreased significantly after a time course of 24 h (Fig 6F), mirroring miR-155 upregulation patterns observed in prior experiments. Collectively, these results demonstrated that miR-155 directly targets TRAF3 and STAT1 in ALV-J-infected CEFs.

### miR-155 represses IFN-I response and facilitates ALV-J replication by dually targeting TRAF3 and STAT1

**Fig 7:**
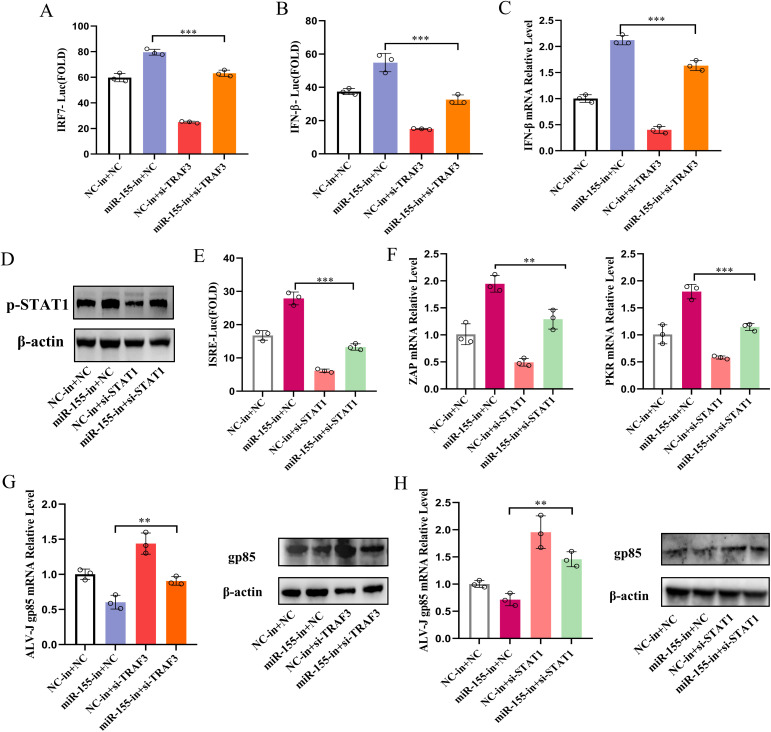
miR-155 represses IFN-β production and signaling and facilitates ALV-J replication by dually targeting TRAF3 and STAT1. **(A-C)** miR-155 inhibits IFN-β production by targeting TRAF3. DF-1 cells were co-transfected with the IRF7 (A) or IFN-β promoter (B) vector, pRL-TK, and si-TRAF3#2 or NC (100 nM), and miR-155-in or NC inhibitor (60 nM), as indicated for 36 h. Cells were then stimulated with poly(I:C) (5 μg/mL) for 12 h, and the luciferase activities were analyzed. (C) CEFs were transfected with NC or si-TRAF3#2 (100 nM) for 12 h, and then infected with ALV-J at an MOI of 1 for 36 h. IFN-β mRNA levels were determined using qRT-PCR. (D-F) miR-155 impairs IFN-I signaling by targeting STAT1. (D and F) CEFs were co-transfected with NC or si-STAT1#2 (100 nM), and miR-155-in or NC inhibitor (60 nM) as indicated for 12 h, and then infected with ALV-J at an MOI of 1 for 36 h. The levels of phosphorylated STAT1 were detected by Western blotting, and ZAP and PKR mRNA levels were determined by qRT-PCR. (E) DF-1 cells were co-transfected with the ISRE reporter vector, pRL-TK, si-STAT1#2 or NC (100 nM) and miR-155-in or NC inhibitor (60 nM) for 36 h, followed by stimulation with IFN-β (200 ng/mL) for 12 h, and luciferase activities were analyzed. **(G and H)** miR-155 targets TRAF3 and STAT1 to enhance ALV-J replication. CEFs were co-transfected with NC, si-TRAF3#2, or si-STAT1#2 (100 nM), and miR-155-in or NC inhibitor (60 nM) as indicated for 12 h. Later, the cells were infected with ALV-J at an MOI of 1 for 36 h, and ALV-J gp85 mRNA and protein levels were determined using qRT-PCR and Western blotting. **, P < 0.01. ***, P < 0.001.

TRAF3 positively controls IFN-I production by activating IRF3 and NF-κB [36]. STAT1 is an essential component of the JAK/STAT signaling cascade, leading to ISRE activation and the production of ISGs [37]. To delineate the roles of TRAF3 and STAT1 in miR-155-mediated suppression of IFN-I responses during ALV-J infection, we silenced these genes in CEFs using siRNA (S9 Fig). TRAF3 downregulation inhibited IFN-β production and impaired IRF7 and IFN-β promoter activity induced by poly(I: C) and suppressed the increased IFN-β production due to miR-155 inhibition (Fig 7A-7C). Similarly, STAT1 silencing attenuated the STAT1 phosphorylation, IFN-β-induced ISRE activity, and ISG production, markedly negating the ability of miR-155 inhibitor to amplify antiviral signaling (Fig 7D-7F). These findings suggested that modulation of IFN-β production and signaling by miR-155 is primarily achieved through targeting the expression of TRAF3 and STAT1.

Afterward, to elaborate on the role of TRAF3 and STAT1 in the miR-155-mediated facilitation of ALV-J replication, knockdown experiments were performed using siRNA in CEFs expressing miR-155 inhibitor to examine their impact on viral replication. TRAF3 and STAT1 downregulation rescued the viral replication inhibited by the miR-155 inhibitor (Fig 7G and 7H). Our data suggested that miR-155 facilitated ALV-J replication by repressing IFN-β production and signaling by targeting TRAF3 and STAT1.

## Discussion

**Fig 8:**
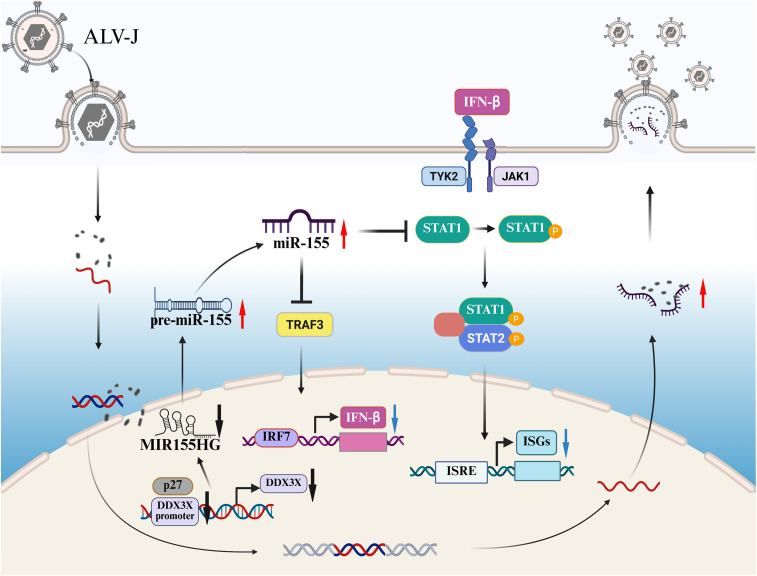
Proposed model for miR-155 activation and its role in the regulation of IFN-I response upon ALV-J infection. ALV-J p27 represses DDX3X expression and promotes MIR155HG processing into mature miR-155. miR-155 directly targets TRAF3 and STAT1, suppressing IFN-β production and signaling, thereby enhancing ALV-J replication. Created in BioRender.com. https://BioRender.com/yz1fjns.

Immune evasion constitutes a fundamental strategy for retroviral persistence [38,39]. IFN-I is the critical mediator in innate antiviral immune responses and has been indicated to inhibit ALV-J replication effectively *in vitro* [40–42]. As a countermeasure, ALV-J targets host proteins involved in IFN-I signaling pathways, thereby interfering with IFN-induced antiviral responses [19,20,43]. Besides, ALV-J exploits miRNA to evade host antiviral surveillance and enhance self-replication [21,22]. However, the precise molecular mechanism by which ALV-J circumvents antiviral immune responses remains incompletely understood. Here, we elucidate a novel miR-155-dependent mechanism through which ALV-J subverts innate immunity, revealing how ALV-J exploitation of miR-155 creates an immune-privileged microenvironment conducive to persistent replication (Fig 8).

The critical role of miR-155 in antiviral immune responses is widely recognized [26]. Previous studies have indicated that miR-155 plays a diverse role in various viral infection models, reflecting the complexity of host-virus interactions. For instance, during VSV and HBV infection, miR-155 promotes IFN-I signaling and suppresses viral replication by targeting SOCS1, a cytokine that negatively regulates IFN signaling [44,45]. However, post-Epstein–Barr virus (EBV) infection, miR-155 modulating NF-κB signaling and suppressing host innate immunity contributes to EBV immortalization [46]. miR-155 negatively regulates antiviral innate responses among HIV-infected progressors [47]. Here, we determined that ALV-J infection significantly promoted the upregulation of miR-155, preventing IFN-β induction and signaling, resulting in the enhancement of ALV-J replication. Nevertheless, it is difficult to verify whether miR-155 is of the high or low contribution of ALV-J in evading antiviral immunity than other reported mechanisms. How different IFN avoidance strategies cooperate during ALV-J infection remains to be determined.

The mechanism involved in activating miR-155 mediated by ALV-J infection was further investigated. miR-155 is the product of the B-cell integration cluster, initially identified as a frequent integration site of ALV-induced lymphomas [48,49]. Insertional activation is an essential pathway for ALV to activate miR-155 expression. However, our previous research suggested that ALV-J-induced activation of miR-155 in non-tumor cells does not result from insertional activation [50]. Virus infections broadly affect miRNA biogenesis at the transcriptional and post-transcriptional levels [51–53]. Here, we demonstrated that ALV-J protein p27, through reduced DDX3X promoter activity, silences DDX3X expression, thereby promoting the processing of MIR155HG into mature miR-155 at the post-transcriptional level. p27 is a group-specific antigen of ALV that is highly conserved among different subgroups. We found that p27-DDX3X interaction and subsequent immune suppression are common mechanisms shared by multiple ALV subgroups (S10 Fig). This represents a broad and conservative mechanism of action for ALVs.

DDX3X, a member of the DEAD-box RNA helicase family, is involved in many RNA metabolism processes, including miRNA processing [54,55]. Our findings provide the first evidence that DDX3X functions as a miRNA regulator in chickens. Our study reveals that through its N-terminal domain, p27 transcriptionally represses DDX3X, thereby reducing MIR155HG stability; the same domain also binds DDX3X. While the concomitant physical interaction between the p27 N-terminal domain and DDX3X suggests a potential mechanistic link, its necessity for DDX3X repression and accumulation of mature miR-155 remains to be definitively established. Future studies creating separation-of-function mutants are required to decouple these processes and elucidate the exact role of this interaction.

TRAF3 and STAT1 constitute core signaling hubs governing IFN-I responses through distinct molecular pathways. TRAF3 acts as a critical adaptor in cytosolic pattern recognition systems (RIG-I-like receptors and TLR pathways), orchestrating IRF3/7 activation to drive IFN-I production [36,56]. Transcription factor STAT1 is central to IFN signaling and contributes to establishing the antiviral state by relaying signals from IFN in the cell nucleus [57,58]. Our findings reveal a dual-pronged immune evasion strategy: miR-155 targeted TRAF3 and STAT1 to inhibit their downstream effectors for eliciting dual effects on IFN expression and signaling to evade the host’s innate antiviral immune response and to establish persistent infection. Our study supplies further evidence for ALV-J evasion of host immune response, and miR-155 is the essential factor mediating this impaired IFN response.

Recent research has indicated that miR-155 is deregulated in multiple cancers and is an oncogene to drive oncogenesis, tumor progression, and metastasis [59–61]. Furthermore, the exploitation of the miR-155 pathway by multiple oncogenic viruses affecting distinct species underscores its central role in oncogenesis. Oncogenic human viruses EBV and KSHV both use the miR-155 pathway to regulate the transformation process, albeit by independent mechanisms. miR-155 is also highly expressed in HTLV-1-positive T-cell lines and is the key molecule involved in the progression of ATLL [29]. Similarly, avian oncogenic viruses, including ALV, MDV, and REV, also utilize miR-155 to drive neoplastic transformation [28,62]. Intriguingly, IFN-I, beyond being best known for its antiviral activity, exhibits tumor-suppressive activity via suppressing tumor cell proliferation and triggering antitumor immune responses [63]. Based on this, it is reasonable to speculate that miR-155 synergistically facilitates both viral persistence and tumor progression in ALV-J pathogenesis. However, this hypothesis needs to be further validated by in vivo tumor models.

In summary, we demonstrated that ALV-J infection significantly activates miR-155 expression. Mechanistically, the interaction of ALV-J p27 protein with DDX3X promotes the processing of MIR155HG into mature miR-155. By dually targeting TRAF3 and STAT1, miR-155 represses IFN production and signaling, thus promoting immune evasion and facilitating ALV-J replication. These findings reveal a novel mechanism by which ALV-J exploits miRNA to escape the host immune response, providing deeper insights into ALV-J pathogenesis.

## Materials and methods

### Ethics statement

Specific pathogen-free (SPF) chicken embryonated eggs were purchased from Jinan Spafas Animal Inc (Spafas, Jinan, China) and incubated in an incubator. All animal experiments were approved by the Shandong Agricultural University Animal Care and Use Committee (permit No. SDAU 23–086; May 7, 2023).

### Cell cultures and virus infection

Primary cultures of chicken embryo fibroblasts (CEF) were extracted from 10-day-old SPF embryos using standard techniques. The DF-1 and HEK293T cells were obtained from the American Type Culture Collection (ATCC, Manassas, VA, USA). The CEF, DF-1, and HEK293T cells were cultured in Dulbecco’s modified Eagle’s medium (DMEM; Hyclone, USA) supplemented with 10% fetal bovine serum (FBS; GIBCO, USA) at 37°C in a humidified atmosphere containing 5% CO_2_. The ALV-J NX0101 strain was maintained in our laboratory. For viral titer assays, ALV-J was serially diluted 10-fold to infect DF-1 cells in 96-well plates, and its infection was determined by immunofluorescent staining to detect ALV-J gp85 protein at 72 hours post-infection (hpi). Titrated viruses were preserved at −80°C until use.

### Reagents and antibodies

Poly(I:C) was purchased from InvivoGen (San Diego, USA). Recombinant Interferon Beta was purchased from USCN (Wuhan, CN). Fludarabine, an inhibitor of STAT1, was obtained from Selleck Chemicals (Houston, USA). The commercialized pRL-TK and pGL4-ISRE-Luc expression plasmids were purchased from Promega (Wisconsin, USA). miR-155 mimics, miR-155 inhibitor, and small interfering RNA (siRNA), as well as their negative control listed in S2 Table, were synthesized by GenePharma (Shanghai, CN). Stat1 (D1K9Y) Rabbit mAb was purchased from Cell Signaling Technology (Danvers, USA). Antibodies against TRAF3 and STAT1 (phospho S727) were purchased from Abcam (Waltham, USA). Antibodies against beta Actin, FLAG, HA, and all secondary antibodies used for immunofluorescence imaging and immunoblotting were purchased from Engibody (Wisconsin, USA). DDX3 Polyclonal antibody, DROSHA Polyclonal antibody, DGCR8 Monoclonal antibody, DICER1 Monoclonal antibody, and AGO2 Monoclonal antibody were purchased from Proteintech (Wuhan, CN). Mouse anti-gp85 antibody was prepared in our laboratory [64].

### Plasmid construction

Plasmids encoding ALV-J Gag, Pol, Env, p10, p15, p19, and p27 (GenBank no. DQ115805.1), p27 ΔN (Δaa 10–143), p27 ΔC (Δaa 153–207), as well as ALV-A p27 (GenBank no. HM452339.1) and ALV-B p27 (GenBank no. HM446005.1) were constructed by cloning the synthesized sequence into pEX-3 (pGCMV/MCS/Neo) with a Flag tag fused to the 3′end. The DDX3X (GenBank no. NM_001030800.2) overexpression vector was constructed by cloning the synthesized sequence into pEX-3 (pGCMV/MCS/Neo) with an HA tag fused to the 3′end. The DDX3X promoter was constructed by cloning the synthesized DDX3X transcription start site -2kb sequence into GPL4-Basic. The chicken IFN-β promoter, IRF7, and NF-κB luciferase reporter were constructed as described previously [65]. The reporter plasmids pmirGLO-TRAF3-wt, pmirGLO-TRAF3-mut, pmirGLO-STAT1-wt, and pmirGLO-STAT1-mut were synthesized by GenePharma (Shanghai, CN).

### Transfection and quantitation real-time PCR

According to the manufacturer’s instructions, Lipofectamine 3000 reagent (Invitrogen) was applied to transfect small RNAs and DNA constructs. Total RNA was isolated using TRIzol reagent (Tiangen, Beijing, CN). For mRNAs, reverse transcription was performed using the FastKing RT kit (with gDNase) (Tiangen, Beijing, CN), and the expression patterns of each gene were determined using TB Green Premix Ex Taq (Takara). For miRNAs, total RNA was reverse-transcribed according to the protocol provided with the miRcute Plus miRNA First-Strand cDNA Kit (Tiangen, Beijing, CN), and the expression analysis of miRNA was executed by using the miRcute miRNA qPCR Detection Kit (Tiangen, Beijing, CN). qPCR was carried out using the Lightcycler 96 (Roche, Sweden). The 2^−ΔΔCt^ method was used to calculate-fold changes in expression relative to Mock or control-treated samples, with U6 or GAPDH levels being used for normalization. All qRT-PCR experiments were performed in triplicate. Primer sequences are listed in S3 Table.

### Co-immunoprecipitation and mass spectrometric assays

The cells were collected and subjected to Co-IP according to the manufacturer’s instructions for Smart-CoIP Co-IP Kit (Wisconsin, USA). Cells were collected on ice-cold lysis buffer and incubated on ice for 20 min with periodic mixing. The cell lysates were centrifuged at 13,000 g for 15 min at 4°C to pellet the cell debris. Pre-clear lysate using the Protein A/G Magnetic Beads. Cell lysates were incubated with the primary antibody and normal IgG at 4°C overnight. Then, a resuspended volume of protein A/G magnetic beads slurry was added to capture the bait-prey immunocomplex. Washed the complex 2–3 times with Wash Buffer, and then eluted with Elution Buffer. The eluted proteins were boiled in 5 × SDS sample buffer (5 min, 100°C) and subjected to SDS-PAGE and immunoblotting.

For mass spectrometry (MS) analysis, the eluted immunoprecipitated products were submitted to the BGI (Shenzhen, CN) mass spectrometry platform for liquid chromatography-tandem MS (LC-MS/MS) analysis.

### RNA Immunoprecipitation (RIP)

Cell pellets were lysed in the RIP buffer supplemented with protease inhibitor and 50 U/ml RNase inhibitor. Each of cell extracts was subjected to RIP with 10 µg rabbit anti-DDX3X or anti-HA and control IgG with Smart-RIP RIP Kit (Engibody, Wisconsin, USA), and the retrieved RNA was subjected to qRT-PCR analysis.

### Western blotting

Cells were lysed in RIPA lysis buffer, and the extracts were then clarified by centrifugation at 13,000 g for 15 min at 4°C. The protein concentration of the lysates was determined by the BCA protein assay (Tiangen, CN). Lysates were incubated in 5 × SDS sample buffer (5 min, 100°C), separated by 10% or 12% SDS-PAGE and transferred onto PVDF membranes. The membrane was blocked with 5% nonfat milk for 1 h, followed by incubation with the indicated primary and secondary antibodies.

### Immunofluorescence and confocal imaging

Cells were grown on 24-well glass coverslips and subjected to the indicated treatments. The cells were fixed with 4% paraformaldehyde for 30 min, permeabilized with 0.2% TritonX-100 for 10 min, and blocked in 5% BSA for 1 h. Next, the cells were incubated with corresponding primary antibodies for overnight at 4°C. Then, this was incubated with Alexa Fluor 488 anti-mouse and Alexa Fluor 594 anti-rabbit lgG H&L for 2 h, and then stained with 4′,6-diamidino-2-phenylindole (DAPI) for 10 min. The samples were detected by the Leica SP2 confocal system (Leica Microsystems, Wetzlar, Germany).

### ELISA

The p27 protein levels in cell cultures were analyzed using Avian Leukosis Virus ELISA Group Specific Antigen Test Kit (NECVB; Harbin, CN) according to the manufacturer’s instructions. The IFN-β protein levels in cell cultures were analyzed using a chicken IFN-β ELISA kit (USCN Life Science; Wuhan, CN) according to the manufacturer’s instructions.

### Luciferase reporter assay

Following lysis in Passive Lysis Buffer, firefly and Renilla luciferase activities were measured using the Dual-Luciferase Reporter Assay System (Promega), according to the manufacturer’s protocol. Relative luciferase activity was normalized to Renilla luciferase activity.

### Statistical analysis

Data are presented as means and standard deviations (±SD) and were analyzed by T-test for two groups or by one-way ANOVA for multiple groups. P values <0.05 were considered statistically significant as indicated in the figure legends. All graphs were made using GraphPad Prism software.

## Supporting information

S1 Fig CEFs possess a complete miRNA processing machinery. **(A)** Western blotting analysis of Drosha, DGCR8, Dicer, and Ago2 expression in CEFs and 293T whole cell lysates. **(B)** Knockdown efficiency of Drosha and Dicer. CEFs were transfected with si-Drosha# 1–3, si-Dicer#1–3, or Negative Control (NC) (100 nM). After 48 h, Drosha and Dicer mRNA and protein levels were determined using qRT-PCR and Western blotting. **(C)** Knockdown of Drosha or Dicer inhibits the expression of miR-155. CEFs were transfected with si-Drosha#1, si-Dicer#1, or NC (100 nM). After 48 h, the miR-155 levels were determined using qRT-PCR. **, P < 0.01. ***, P < 0.001. ns, P > 0.05.
(TIF)

S2 Fig Illustration of the complete structure of p27 and its truncated variants. Schematic diagram of the complete structure of p27 and its truncated forms. ΔN, N-terminal deletion; ΔC, C-terminal deletion.
(TIF)

S3 Fig p27 interacts with DDX3X in both cytoplasmic and nuclear fractions. CEFs were transfected with the p27-FLAG plasmid for 48 h, and then cytoplasmic and nuclear proteins were isolated for immunoprecipitation. Protein interactions were analyzed by immunoblotting using the indicated antibodies. Mouse IgG antibodies were utilized as a control.
(TIF)

S4 Fig p27 N-terminal domain interacts with DDX3X. CEFs were transfected with p27 ΔN or p27 ΔC plasmid for 48 h, followed by coimmunoprecipitation and immunoblot analysis were performed with the indicated antibodies. Mouse IgG antibodies were utilized as a control.
(TIF)

S5 Fig p27 does not affect DDX3X mRNA and protein stability but inhibits DDX3X promoter activity through the N-terminal domain. **(A)** p27 does not affect DDX3X mRNA stability. CEFs were transfected with p27-FLAG plasmid or the corresponding control for 24h, then treated with the transcription inhibitor actinomycin D (5 μg/mL). DDX3X mRNA was quantified by qRT-PCR at 0, 3, 6, and 9 h. **(B)** p27 does not affect DDX3X protein stability. CEFs were transfected with p27-FLAG or the corresponding control for 24 h, then treated with the proteasome inhibitor MG132 (10 µM), the autophagy inhibitor CQ (25µM), and the apoptosis inhibitor Z-VAD-FMK (10 µM). Cell lysates were analyzed by Western blotting after 12h. **(C)** p27 N-terminal domain inhibits DDX3X promoter activity. The DDX3X promoter along with pRL-TK were co-transfected with the designated plasmid into DF-1 cells for 48 h. Then, cells were harvested for the luciferase assay. **, P < 0.01. ns, P > 0.05.
(TIF)

S6 Fig Overexpression and knockdown efficiency of DDX3X. **(A)** CEFs were transfected with DDX3X-HA plasmid or control plasmid for 48 h. DDX3X mRNA and protein levels were determined using qRT-PCR and Western blotting. **(B)** CEFs were transfected with NC or si-DDX3X#1–3. After 48 h, DDX3X mRNA and protein levels were determined using qRT-PCR and Western blotting. *, P < 0.05. ***, P < 0.001. ns, P > 0.05.
(TIF)

S7 Fig p27-DDX3X moderately regulates expression of miR-146a-5p, Let-7b, and miR-221. **(A-C)** CEFs were transfected with p27-FLAG plasmid, DDX3X-HA plasmid, or both for 48 h, and then the miR-146a-5p (A), Let-7b (B), and miR-221 (C) levels were measured using qRT-PCR. *, P < 0.05. **, P < 0.01. ns, P > 0.05.
(TIF)

S8 Fig Knockdown of DDX3X reduces the stability of MIR155HG and increases the accumulation of mature 155. **(A)** MIR155HG stability assay. CEFs were transfected with si-DDX3X#3 or NC (100 nM) for 24 h, then treated with the transcription inhibitor actinomycin D (5 μg/mL). MIR155HG was quantified by qRT-PCR at 0, 3, 6, and 9 h. **(B)** CEFs were transfected with si-DDX3X#3 or NC (100 nM) for 12 h, followed by infection with ALV-J for 36 h, and the mRNA expression of miR-155, pre-miR-155, and MIR155HG were analyzed by qRT-PCR. *, P < 0.05. **, P < 0.01. ***, P < 0.001.
(TIF)

S9 Fig Knockdown efficiency of TRAF3 and STAT1. **(A and B)** CEFs were transfected with NC, si-TRAF3#1–3, or si-STAT1#1–3 (100 nM). After 48 h, TRAF3 and STAT1 mRNA and protein levels were determined using qRT-PCR and Western blotting. **, P < 0.01. ns, P > 0.05.
(TIF)

S10 Fig p27-DDX3X-miR-155 axis is conserved across ALV subgroups. **(A)** ALV-A p27 suppresses DDX3X expression and activates miR-155. CEFs were transfected with the indicated plasmids for 48 h. The DDX3X mRNA and protein levels were measured using qRT-PCR and Western blotting. The miR-155 levels were measured using qRT-PCR. **(B)** ALV-B p27 represses DDX3X expression and activates miR-155. CEFs were transfected with the indicated plasmids for 48 h. The DDX3X mRNA and protein levels were measured using qRT-PCR and Western blotting. The miR-155 levels were measured using qRT-PCR. **(C)** ALV-A and ALV-B p27 interact with DDX3X. CEFs were transfected with ALV-A or ALV-B p27 for 48 h followed by coimmunoprecipitation and immunoblot analysis with the indicated antibodies. **(D)** ALV-A enhances miR-155 expression and suppresses the induction of IFN-β and ISGs. CEFs were infected with ALV-A at an MOI of 1 for 48 h. The transcription levels of miR-155, IFN-β, ZAP, and PKR were measured using qRT-PCR. *, P < 0.05. **, P < 0.01. ***, P < 0.001.
(TIF)

S1 Table MS data of selected proteins. (DOCX)

S2 Table Sequences of miRNA mimics, inhibitors, and siRNA. (DOCX)

S3 Table Primers used in qRT-PCR analysis. (DOCX)

S1 Data Excel spreadsheet containing, in separate sheets, the underlying numerical data for Figs 1A-1K, 2A-2D, 2G-2L, 3A-3D, 4B, 4D, 4E, 5A, 5C, 6B, 6C, 7A-7C, and 7E-7H and S1B, S1C, S5A, S5C, S6A, S6B, S7A-S7C, S8A, S8B, S9A, S9B, S10A, S10B, and S10D. (XLSX)

S1 Raw images Raw Western blotting data. (PDF)
