# Supplementary figures and images for: Avian leukosis virus subgroup J evades innate immunity by activating miR-155 to dually target TRAF3 and STAT1
Source: PLoS Pathog. 2025 Oct 9;21(10):e1013552. doi: 10.1371/journal.ppat.1013552 (PMC12510514; doi:10.1371/journal.ppat.1013552)

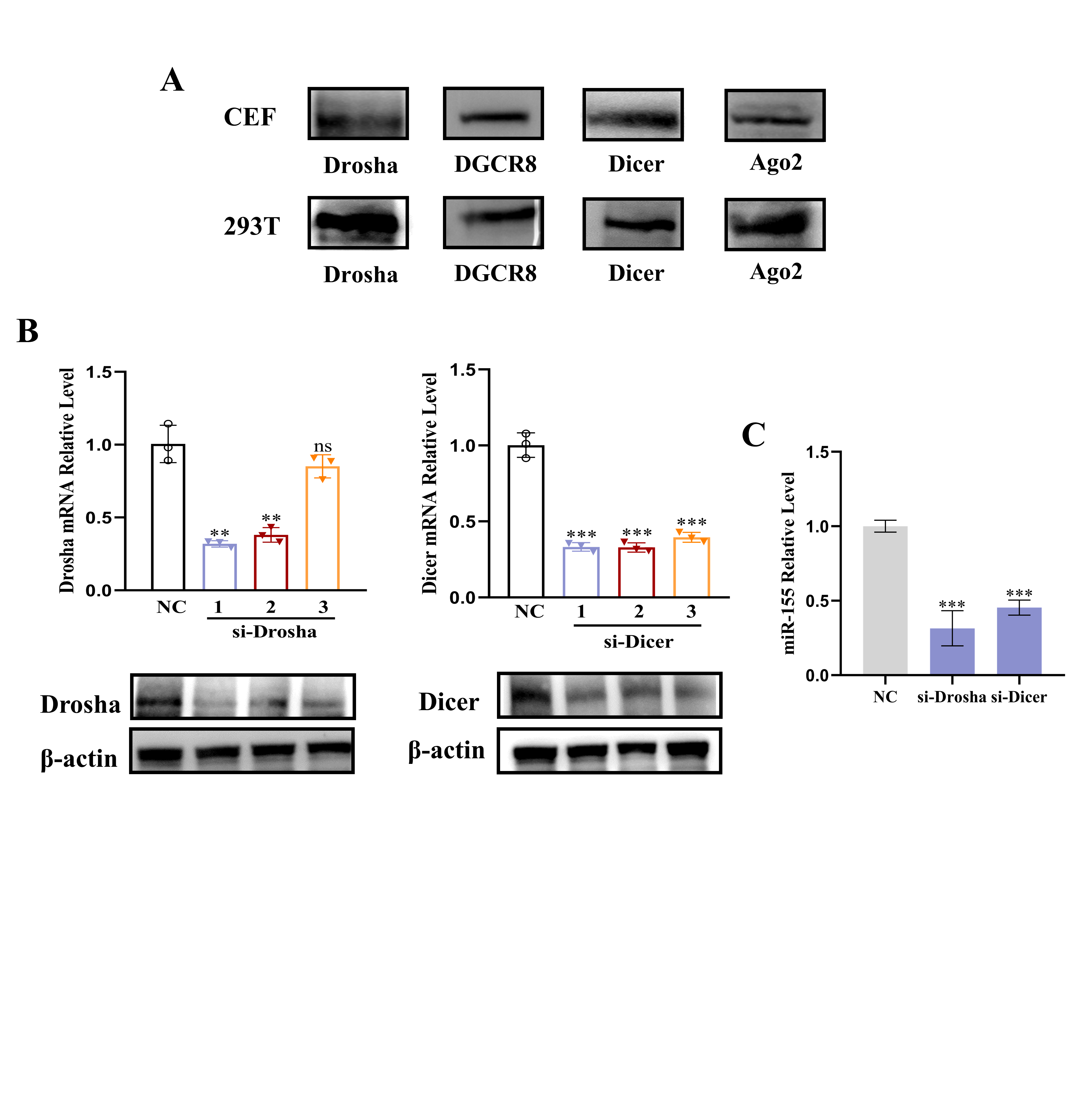

Supplement: S1 Fig — (A) Western blotting analysis of Drosha, DGCR8, Dicer, and Ago2 expression in CEFs and 293T whole cell lysates. (B) Knockdown efficiency of Drosha and Dicer. CEFs were transfected with si-Drosha# 1–3, si-Dicer#1–3, or Negative Control (NC) (100 nM). After 48 h, Drosha and Dicer mRNA and protein levels were determined using qRT-PCR and Western blotting. (C) Knockdown of Drosha or Dicer inhibits the expression of miR-155. CEFs were transfected with si-Drosha#1, si-Dicer#1, or NC (100 nM). After 48 h, the miR-155 levels were determined using qRT-PCR. **, P < 0.01. ***, P < 0.001. ns, P > 0.05. (TIF) [file ppat.1013552.s001.tif]

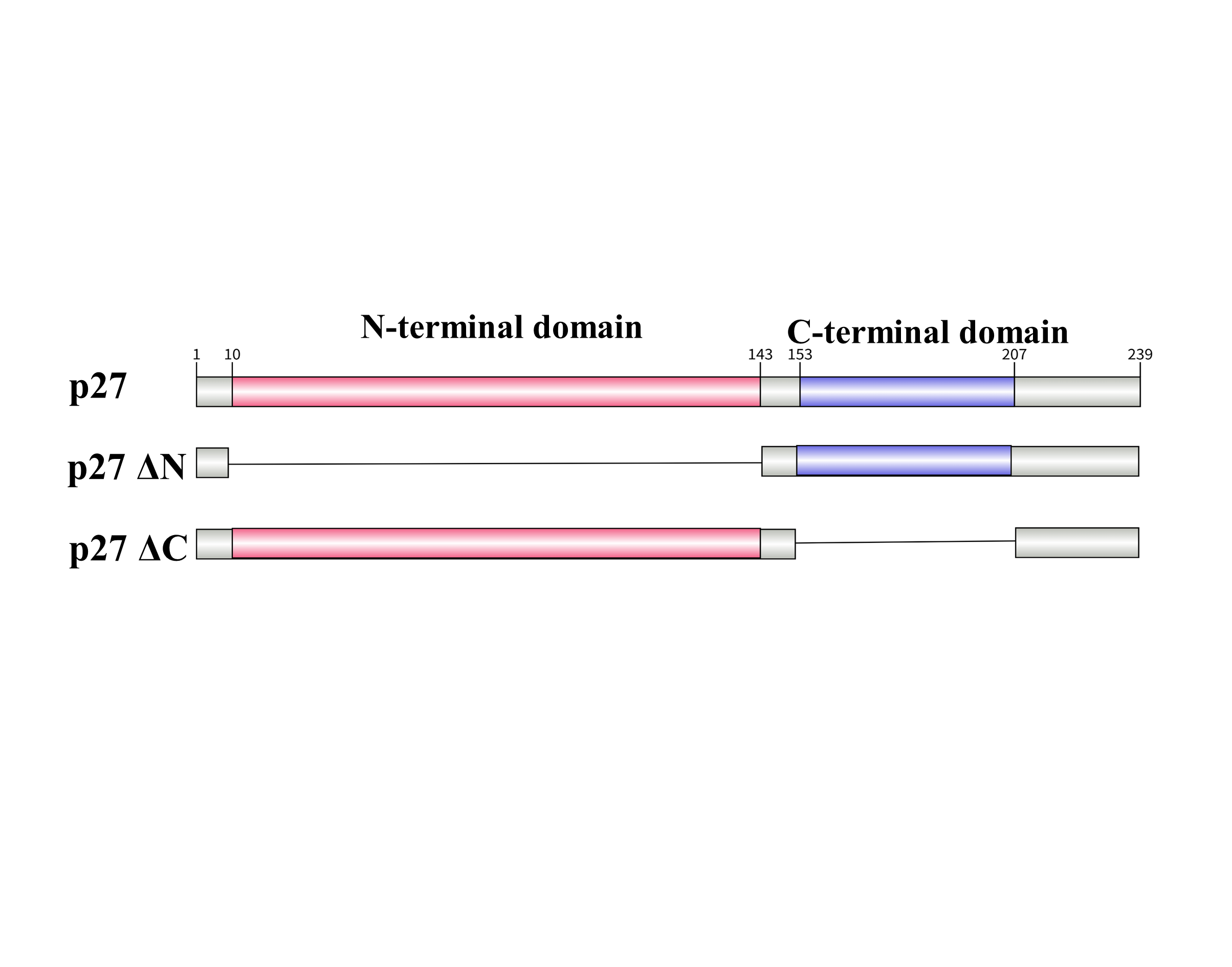

Supplement: S2 Fig — Schematic diagram of the complete structure of p27 and its truncated forms. ΔN, N-terminal deletion; ΔC, C-terminal deletion. (TIF) [file ppat.1013552.s002.tif]

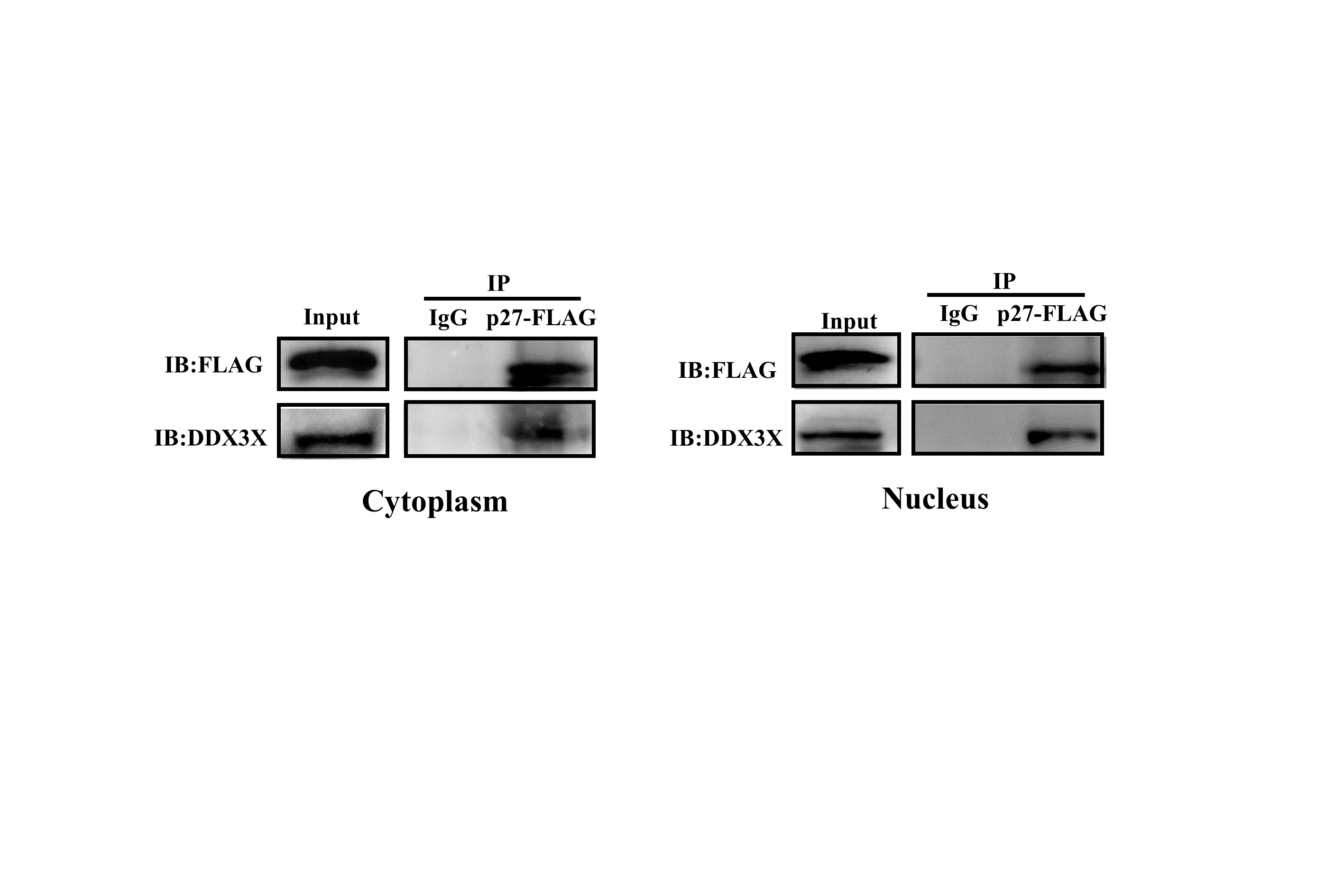

Supplement: S3 Fig — CEFs were transfected with the p27-FLAG plasmid for 48 h, and then cytoplasmic and nuclear proteins were isolated for immunoprecipitation. Protein interactions were analyzed by immunoblotting using the indicated antibodies. Mouse IgG antibodies were utilized as a control. (TIF) [file ppat.1013552.s003.tif]

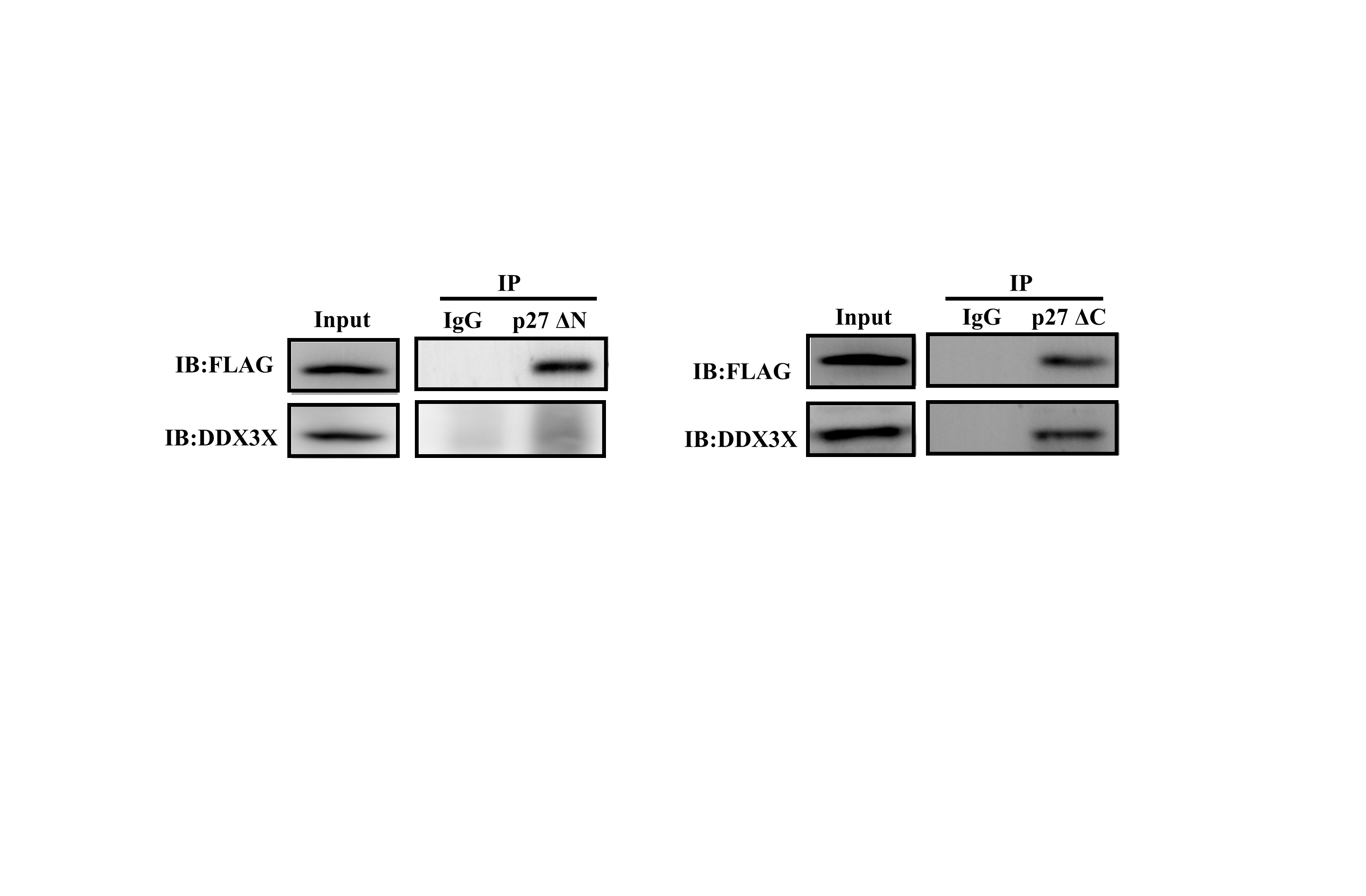

Supplement: S4 Fig — CEFs were transfected with p27 ΔN or p27 ΔC plasmid for 48 h, followed by coimmunoprecipitation and immunoblot analysis were performed with the indicated antibodies. Mouse IgG antibodies were utilized as a control. (TIF) [file ppat.1013552.s004.tif]

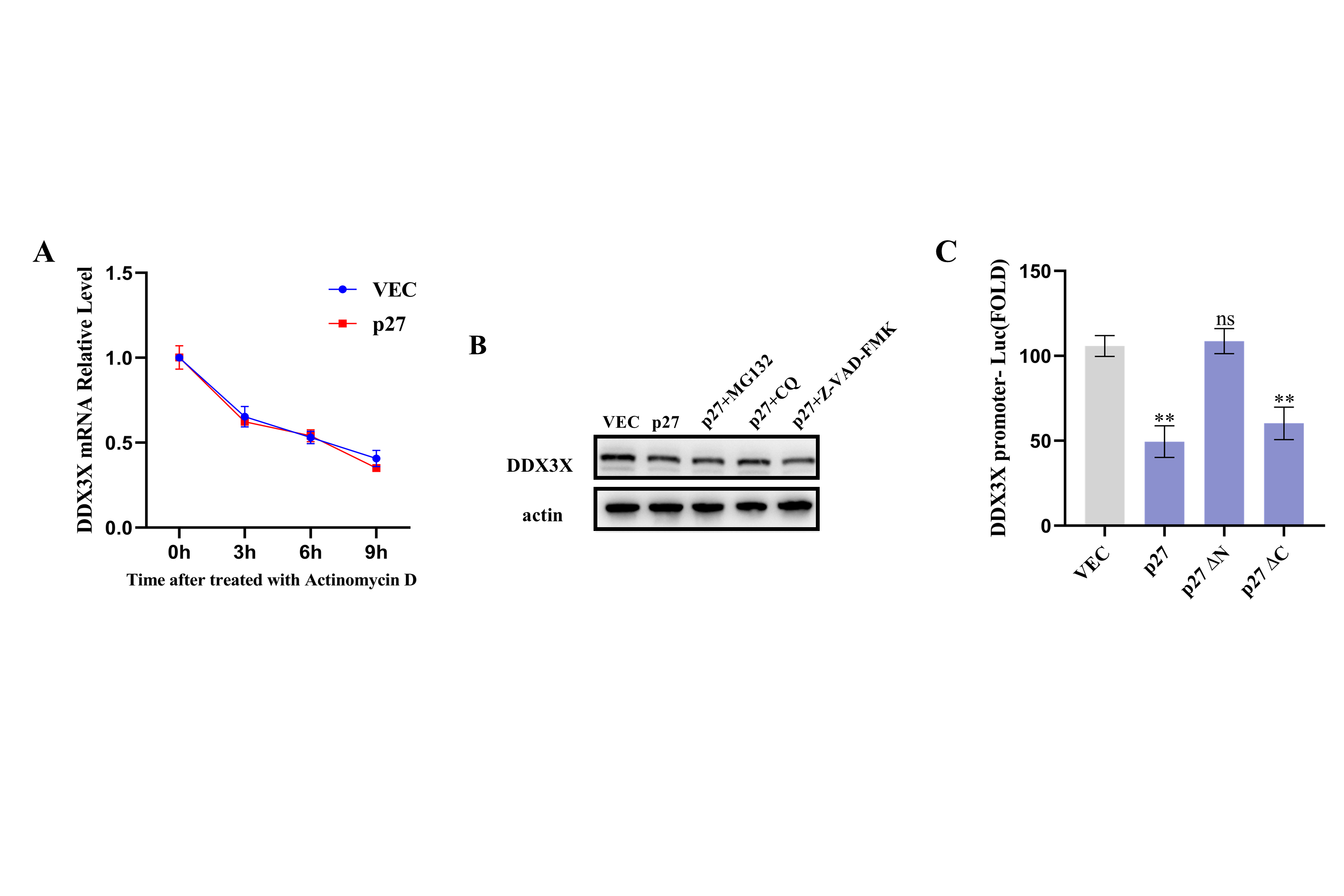

Supplement: S5 Fig — (A) p27 does not affect DDX3X mRNA stability. CEFs were transfected with p27-FLAG plasmid or the corresponding control for 24h, then treated with the transcription inhibitor actinomycin D (5 μg/mL). DDX3X mRNA was quantified by qRT-PCR at 0, 3, 6, and 9 h. (B) p27 does not affect DDX3X protein stability. CEFs were transfected with p27-FLAG or the corresponding control for 24 h, then treated with the proteasome inhibitor MG132 (10 µM), the autophagy inhibitor CQ (25µM), and the apoptosis inhibitor Z-VAD-FMK (10 µM). Cell lysates were analyzed by Western blotting after 12h. (C) p27 N-terminal domain inhibits DDX3X promoter activity. The DDX3X promoter along with pRL-TK were co-transfected with the designated plasmid into DF-1 cells for 48 h. Then, cells were harvested for the luciferase assay. **, P < 0.01. ns, P > 0.05. (TIF) [file ppat.1013552.s005.tif]

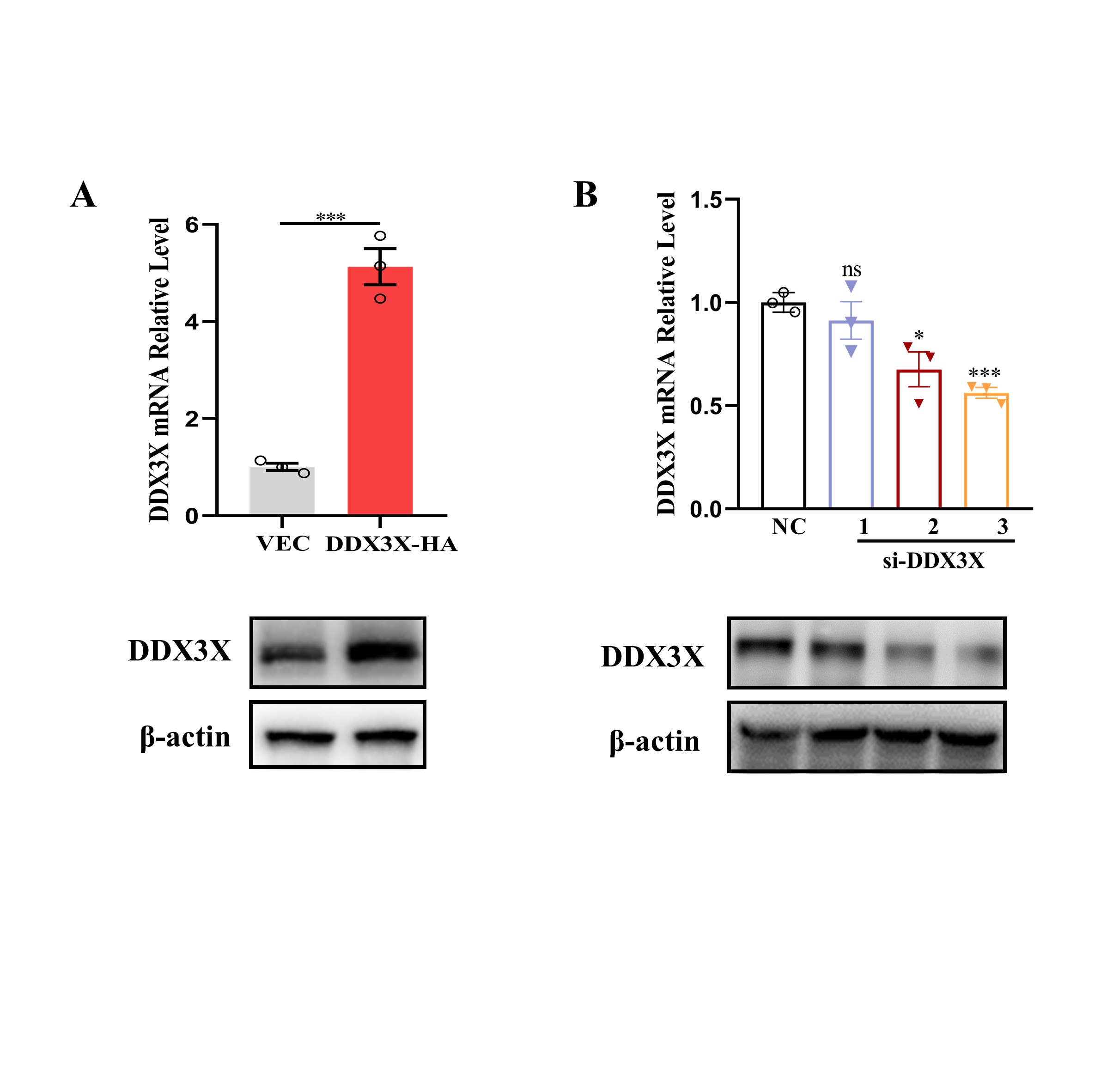

Supplement: S6 Fig — (A) CEFs were transfected with DDX3X-HA plasmid or control plasmid for 48 h. DDX3X mRNA and protein levels were determined using qRT-PCR and Western blotting. (B) CEFs were transfected with NC or si-DDX3X#1–3. After 48 h, DDX3X mRNA and protein levels were determined using qRT-PCR and Western blotting. *, P < 0.05. ***, P < 0.001. ns, P > 0.05. (TIF) [file ppat.1013552.s006.tif]

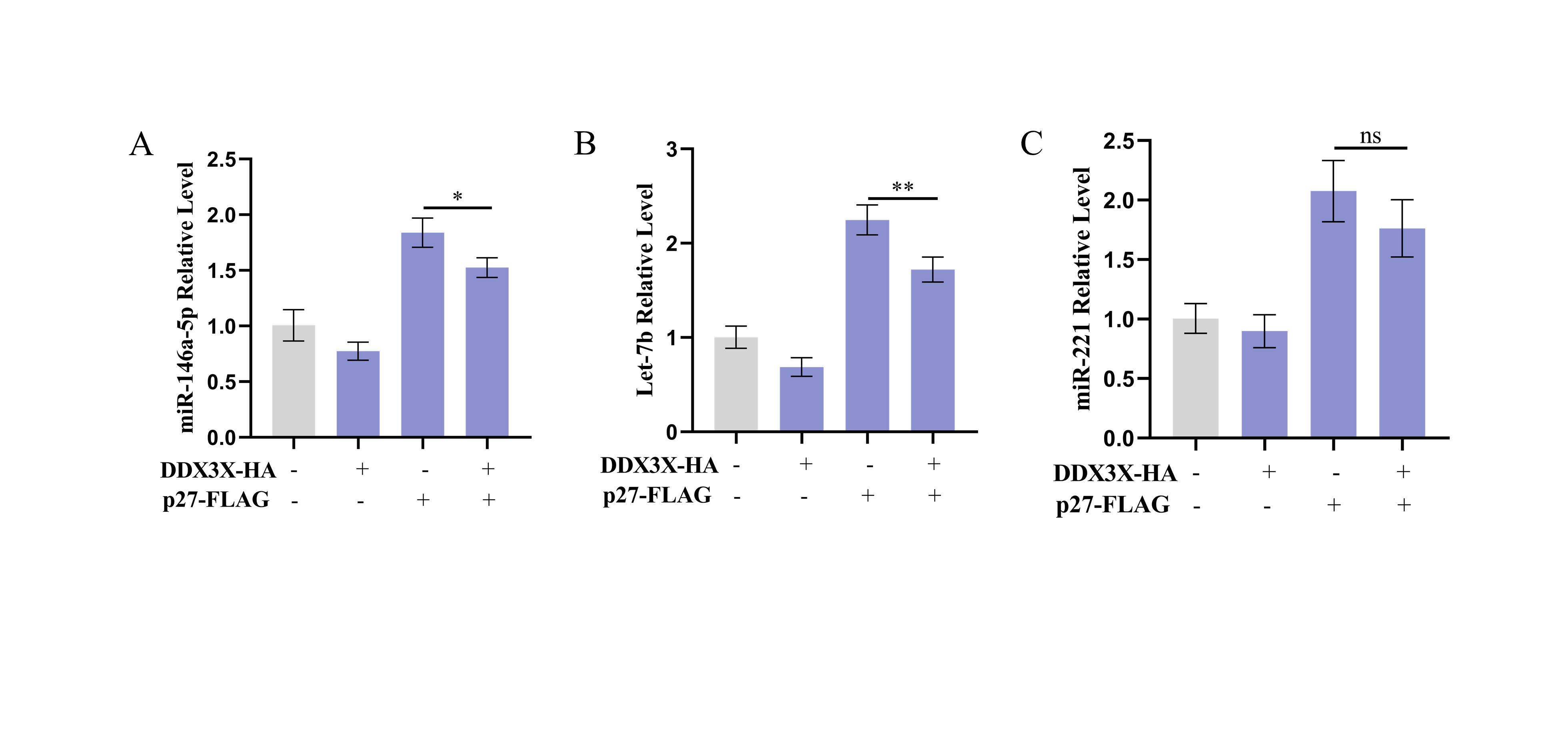

Supplement: S7 Fig — (A-C) CEFs were transfected with p27-FLAG plasmid, DDX3X-HA plasmid, or both for 48 h, and then the miR-146a-5p (A), Let-7b (B), and miR-221 (C) levels were measured using qRT-PCR. *, P < 0.05. **, P < 0.01. ns, P > 0.05. (TIF) [file ppat.1013552.s007.tif]

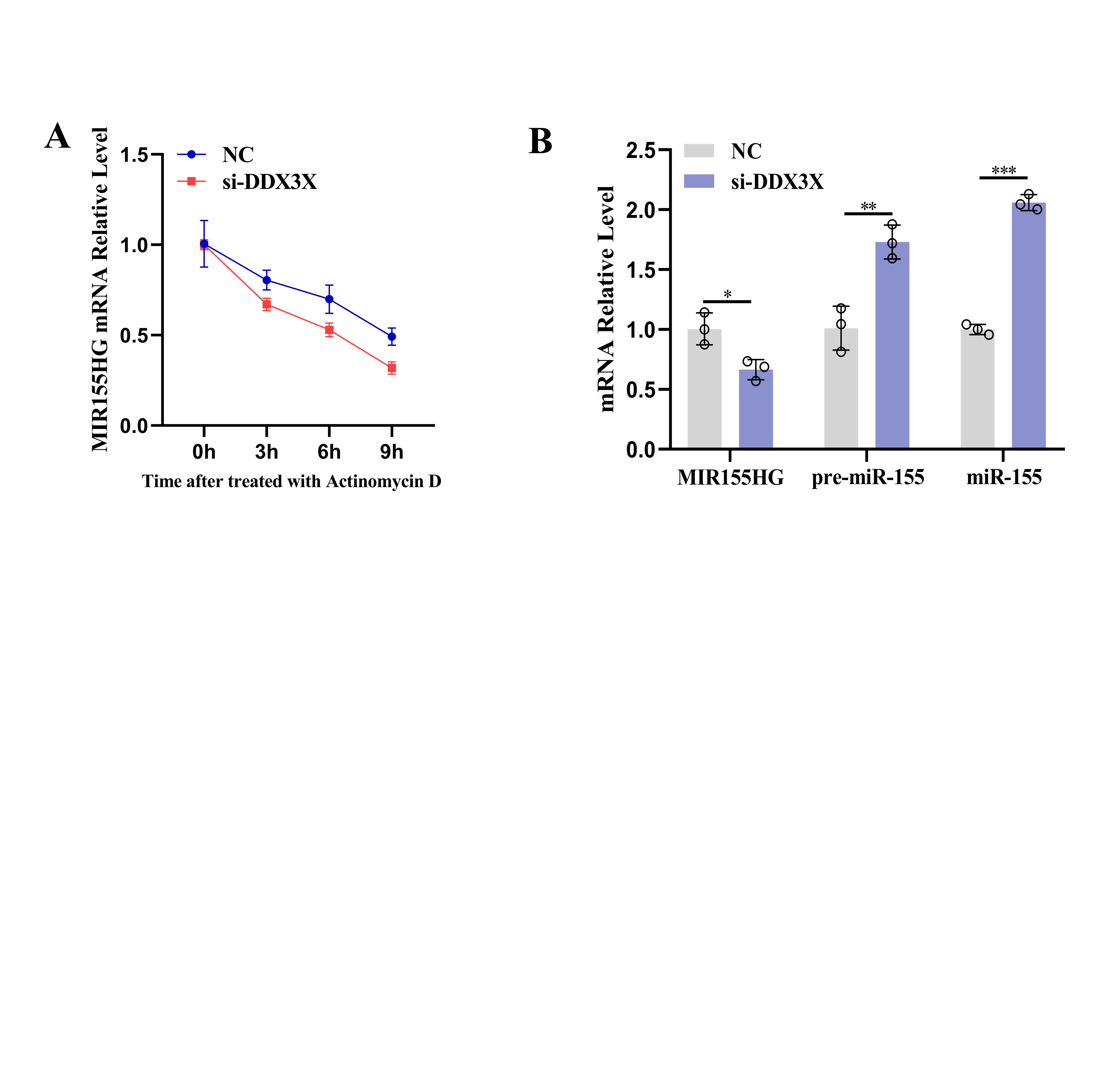

Supplement: S8 Fig — (A) MIR155HG stability assay. CEFs were transfected with si-DDX3X#3 or NC (100 nM) for 24 h, then treated with the transcription inhibitor actinomycin D (5 μg/mL). MIR155HG was quantified by qRT-PCR at 0, 3, 6, and 9 h. (B) CEFs were transfected with si-DDX3X#3 or NC (100 nM) for 12 h, followed by infection with ALV-J for 36 h, and the mRNA expression of miR-155, pre-miR-155, and MIR155HG were analyzed by qRT-PCR. *, P < 0.05. **, P < 0.01. ***, P < 0.001. (TIF) [file ppat.1013552.s008.tif]

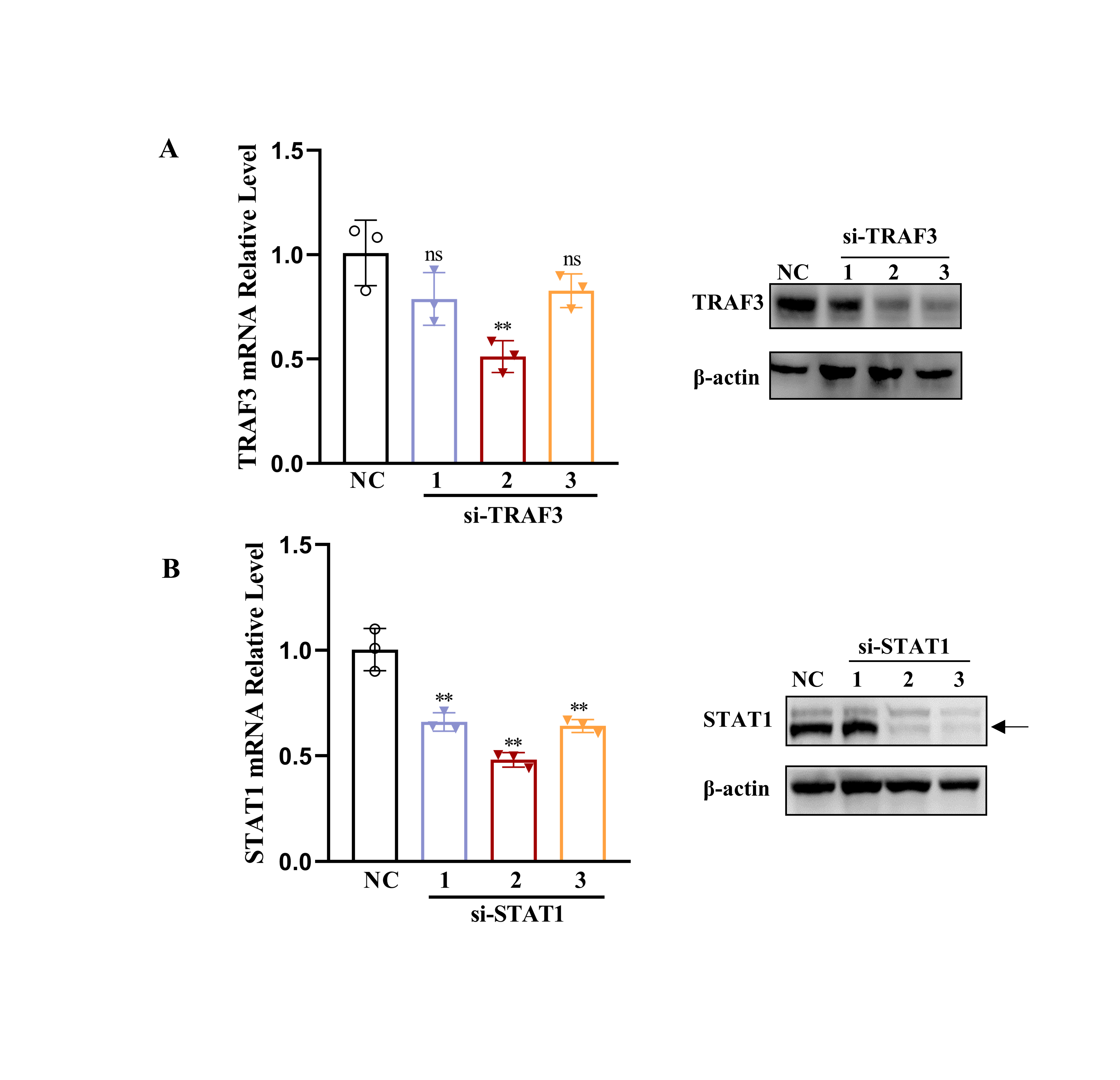

Supplement: S9 Fig — (A and B) CEFs were transfected with NC, si-TRAF3#1–3, or si-STAT1#1–3 (100 nM). After 48 h, TRAF3 and STAT1 mRNA and protein levels were determined using qRT-PCR and Western blotting. **, P < 0.01. ns, P > 0.05. (TIF) [file ppat.1013552.s009.tif]

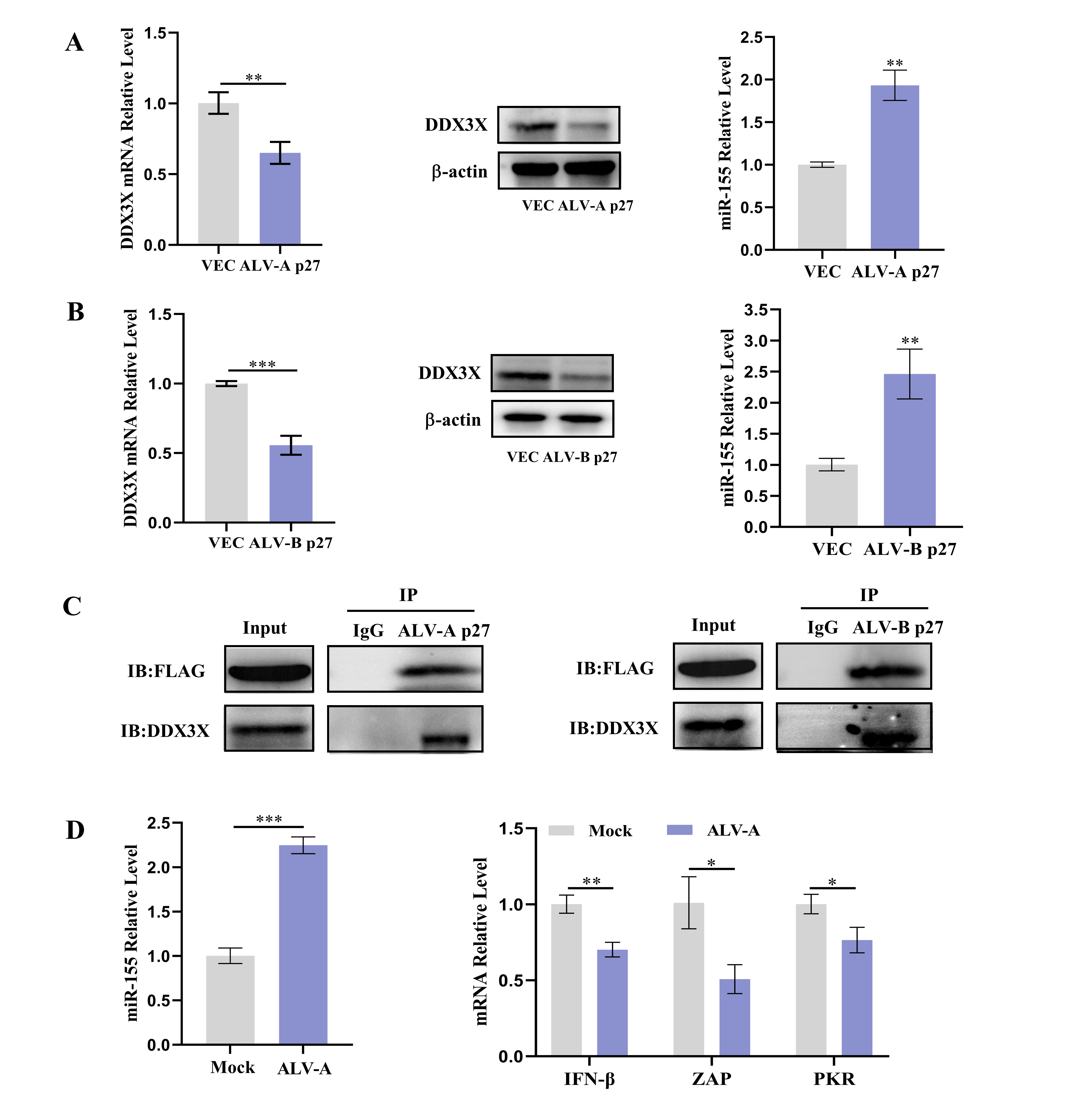

Supplement: S10 Fig — (A) ALV-A p27 suppresses DDX3X expression and activates miR-155. CEFs were transfected with the indicated plasmids for 48 h. The DDX3X mRNA and protein levels were measured using qRT-PCR and Western blotting. The miR-155 levels were measured using qRT-PCR. (B) ALV-B p27 represses DDX3X expression and activates miR-155. CEFs were transfected with the indicated plasmids for 48 h. The DDX3X mRNA and protein levels were measured using qRT-PCR and Western blotting. The miR-155 levels were measured using qRT-PCR. (C) ALV-A and ALV-B p27 interact with DDX3X. CEFs were transfected with ALV-A or ALV-B p27 for 48 h followed by coimmunoprecipitation and immunoblot analysis with the indicated antibodies. (D) ALV-A enhances miR-155 expression and suppresses the induction of IFN-β and ISGs. CEFs were infected with ALV-A at an MOI of 1 for 48 h. The transcription levels of miR-155, IFN-β, ZAP, and PKR were measured using qRT-PCR. *, P < 0.05. **, P < 0.01. ***, P < 0.001. (TIF) [file ppat.1013552.s010.tif]
