# Supplementary material for: Avian leukosis virus subgroup J evades innate immunity by activating miR-155 to dually target TRAF3 and STAT1
Source: PLoS Pathog. 2025 Oct 9;21(10):e1013552. doi: 10.1371/journal.ppat.1013552 (PMC12510514; doi:10.1371/journal.ppat.1013552)
Supplement: S1 Table — (DOCX) [file ppat.1013552.s011.docx]

**S1 Table.** MS data of selected proteins

| Gene | Description | Accession | Gene Ontology |
| --- | --- | --- | --- |
| DDX3X | RNA helicase | A0A3Q2U0S7 | RNA Metabolism |
| KTN1 | Kinectin | Q90631 | kinesin binding |
| RPL3 | Large ribosomal subunit protein uL3 | Q5ZJZ2 | proteins synthesis |
| EEF1 | Eukaryotic translation elongation factor 1 | Q6EE30 | proteins synthesis |
| EEF1B2 | Elongation factor 1-beta | F1NYA9 | proteins synthesis |
