# Supplementary material for: Avian leukosis virus subgroup J evades innate immunity by activating miR-155 to dually target TRAF3 and STAT1
Source: PLoS Pathog. 2025 Oct 9;21(10):e1013552. doi: 10.1371/journal.ppat.1013552 (PMC12510514; doi:10.1371/journal.ppat.1013552)
Supplement: S2 Table — (DOCX) [file ppat.1013552.s012.docx]

**S2 table.** Sequences of miRNA mimics, inhibitors, and siRNA

| Small RNA | Sequance (5′-3′) |
| --- | --- |
| miR-155 mimics  miR-155 inhibitor  si-STAT1#1  si-STAT1#2  si-STAT1#3  si-TRAF3#1  si-TRAF3#2  si-TRAF3#3  si-DDX3X#1  si-DDX3X#2  si-DDX3X#3  si-Drosha#1  si-Drosha#2  si-Drosha#3  si-Dicer#1  si-Dicer#2  si-Dicer#3 | UUAAUGCUAAUCGUGAUAGGGG  CCCCUAUCACGAUUAGCAUUAA  GGAUGUGCAAGACGAAUAUTT  GGAGCAGAAACUUACUUAUTT  GGCACUUGCAACUGAAAGATT  GUGGAGGAUAAGUAUAAAUTT  GUGCCAGAGUUUACCUUAATT  GGCCAUUUAAACAGAAAGUTT  CAGGUCGACUGGUUGAUAUTT  GCCGUAAGCAAUAUCCAAUTT  CCACCUCAUUCUUCAAUGATT  GGGUAUUCCCAGCAGUCAUTT  GUCGCCUCAUCAUAGAAGUTT  GCCCCAGAUUUCACUUCAUTT  GAGCUGUCCUAUCAGAUCATT  GAGAACAGUCCAAGAGUUATT  GACCAAAUGUCUGUGAGUUTT |
