# Supplementary material for: Avian leukosis virus subgroup J evades innate immunity by activating miR-155 to dually target TRAF3 and STAT1
Source: PLoS Pathog. 2025 Oct 9;21(10):e1013552. doi: 10.1371/journal.ppat.1013552 (PMC12510514; doi:10.1371/journal.ppat.1013552)
Supplement: S3 Table — (DOCX) [file ppat.1013552.s013.docx]

**S3 Table.** Primers used in qRT-PCR analysis

| Primers | Sequance (5′-3′) |
| --- | --- |
| IFN-β-F  IFN-β-R  ZAP-F  ZAP-R  PKR-F  PKR-R  GAPDH-F  GAPDH-R  U6-F  U6-R  miR-155-F  pre-miR-155-F  pre-miR-155-R  MIR155HG-F  MIR155HG-R  miR-146a-5p-F  Let-7b-F  miR-221-F  DDX3X-F  DDX3X-R  ALV-J gp85-F  ALV-J gp85-R  TRAF3-F  TRAF3-R  STAT1-F  STAT1-R  Drosha-F  Drosha-R  Dicer-F  Dicer-R | GCCTCCAGCTCCTTCAGAATACG  CTGGATCTGGTTGAGGAGGCTGT ACCAGTGCTGAGAACAAA  CATCAGGAAGGAGGAAAG ATCTCCTCTACCTGCGGATG GGGTCTCCGGTACGGTTTAT  GAACATCATCCCAGCGTCCA CGGCAGGTCAGGTCAACAAC  CTCGCTTCGGCAGCACA  AACGCTTCACGAATTTGCGT  GGTTAATGCTAATCGTGATAGGG  GGCTAATCGTGATAGGGGTT  GGCGACTCCTACATGTTAGCA  CTTCTGTAGGCTGTATGTTGTTAATGC  TTGTTCCTTGTGAGTTCTGATGAGAG  GTGAGAACTGAATTCCATGGGTT  TGAGGTAGTAGGTTGTGTGGTT  AACCTGGCATACAATGTAGATTTCTGT  GCTCCAACTAGAGAACTCGCTGTG  CGGATCTGCTGCCCAATGTCTG  TGCGTGCGTGGTTATTATTTC  AATGGTGAGGTCGCTGACTGT  ATGAAGAGCAGTGTGGAAT  AACTTGGAATCGGAGGTC  CACGCAGCCAACAACGTATC  TGCCATGTGTATCGGGTCTT  CCATGGATCAAGTGGGGGAC  GCTGCTGTCATCATCCGAGT  ACGGCCAAGTTTAGTCCTGC  TCAGCTGTTTGGGACCTGAG |
