# Supplementary material for: Avian leukosis virus subgroup J evades innate immunity by activating miR-155 to dually target TRAF3 and STAT1
Source: PLoS Pathog. 2025 Oct 9;21(10):e1013552. doi: 10.1371/journal.ppat.1013552 (PMC12510514; doi:10.1371/journal.ppat.1013552)
Supplement: S1 Raw images — (PDF) [file ppat.1013552.s015.pdf]

Figure 2

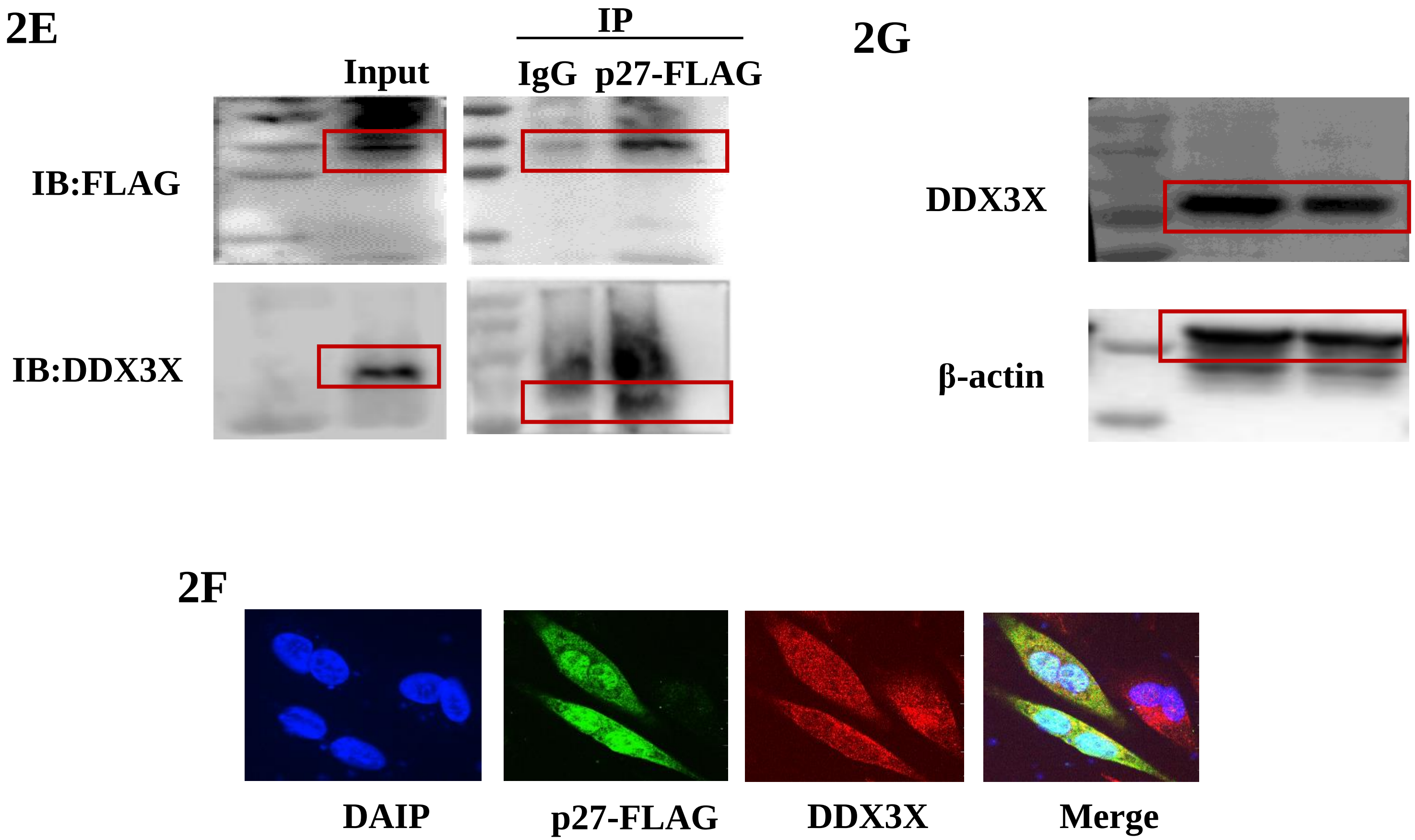

2E
IP
Input
IgG
p27-FLAG
IB:FLAG
IB:DDX3X
2G
DDX3X
β-actin
2F
DAIP
p27-FLAG
DDX3X
Merge

Figure 4

4A

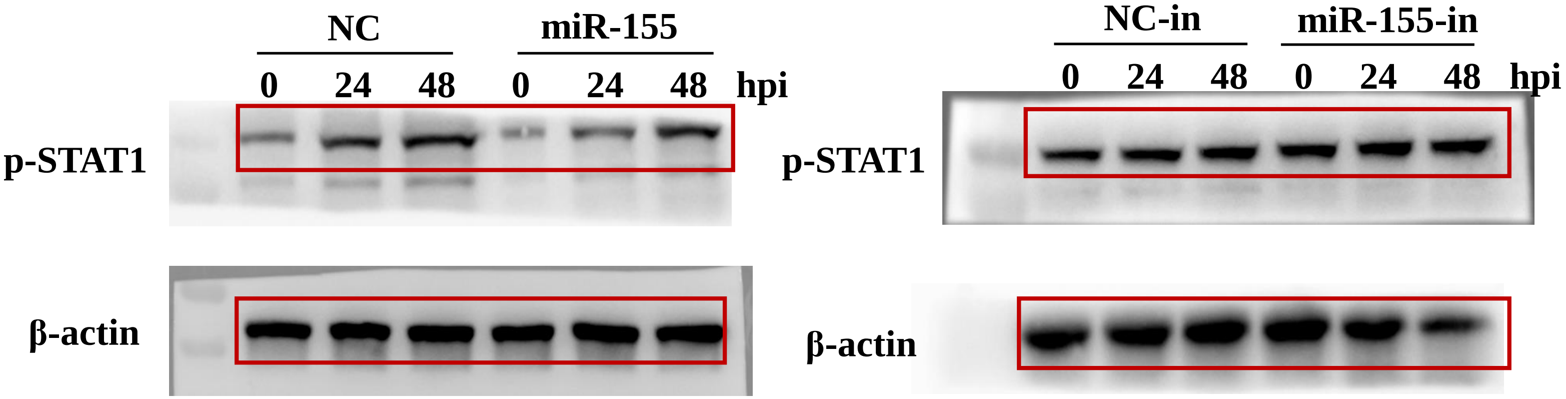

4C

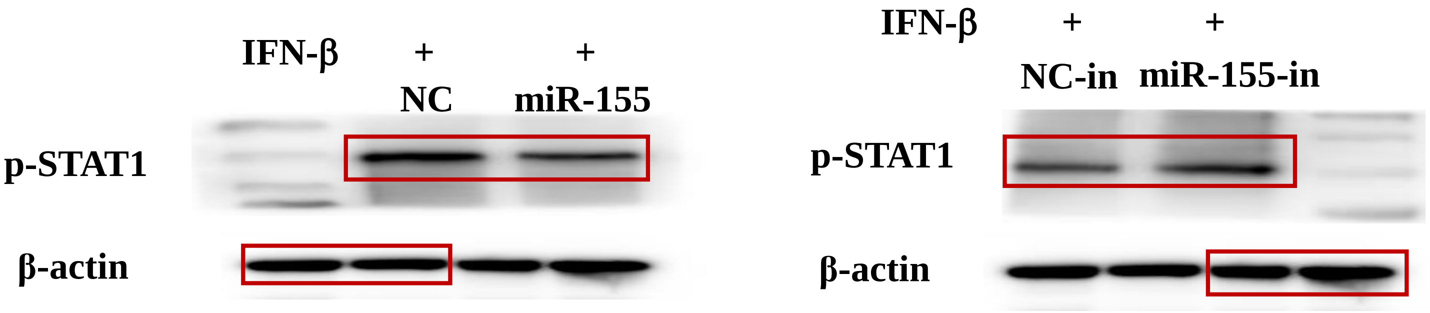

Figure 5

5B

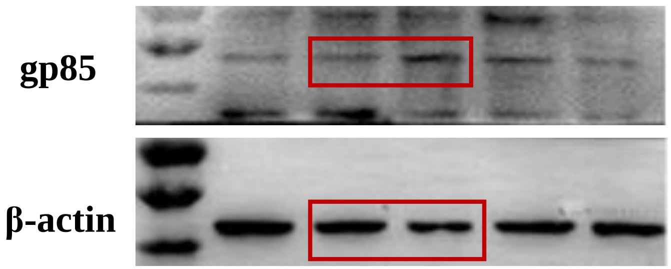

5D

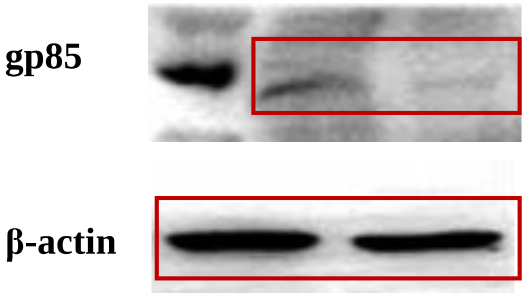

Figure 6

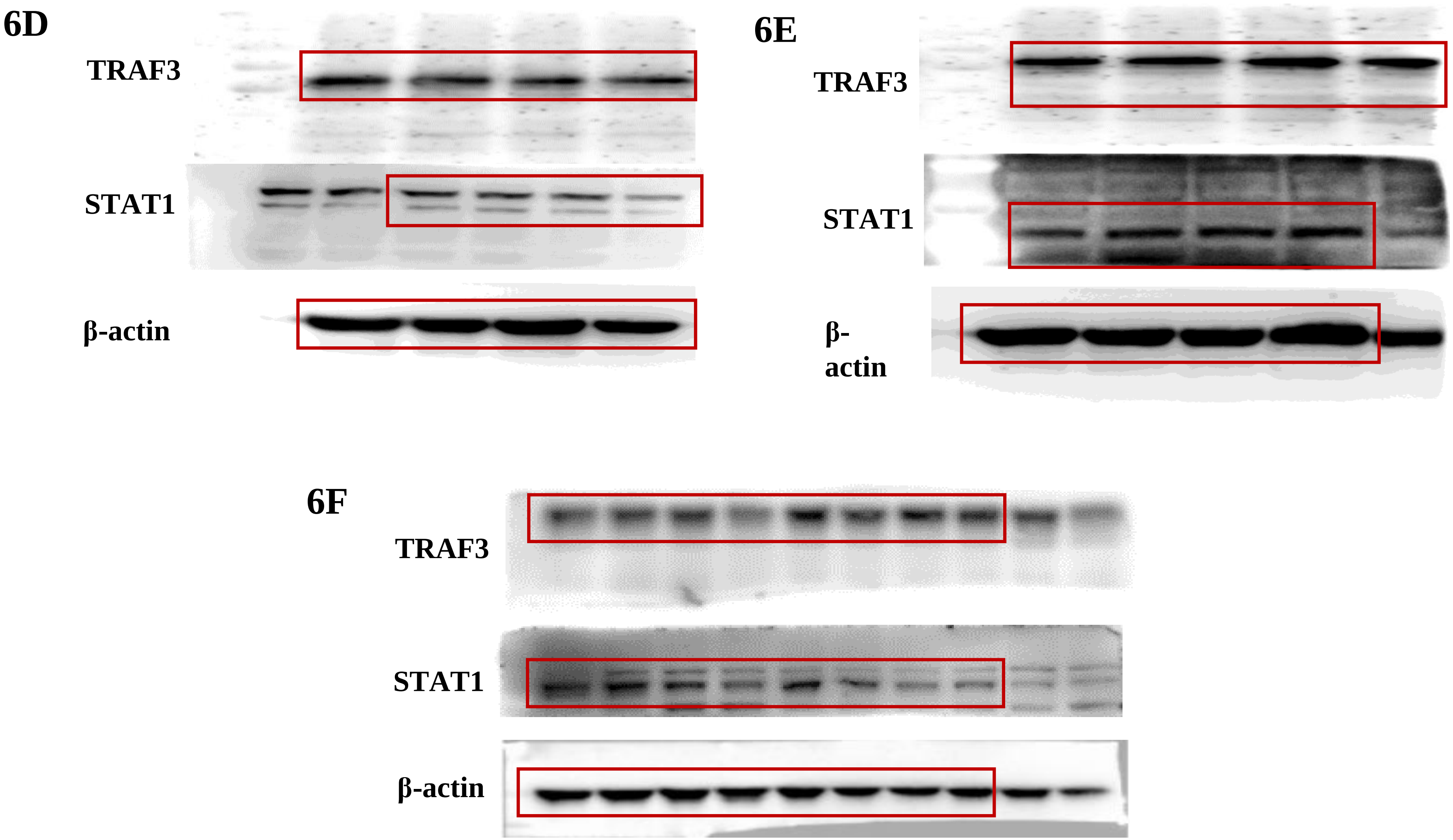
6D
TRAF3
STAT1
β-actin
6E
TRAF3
STAT1
β-
actin
6F
TRAF3
STAT1
β-actin

Figure 7

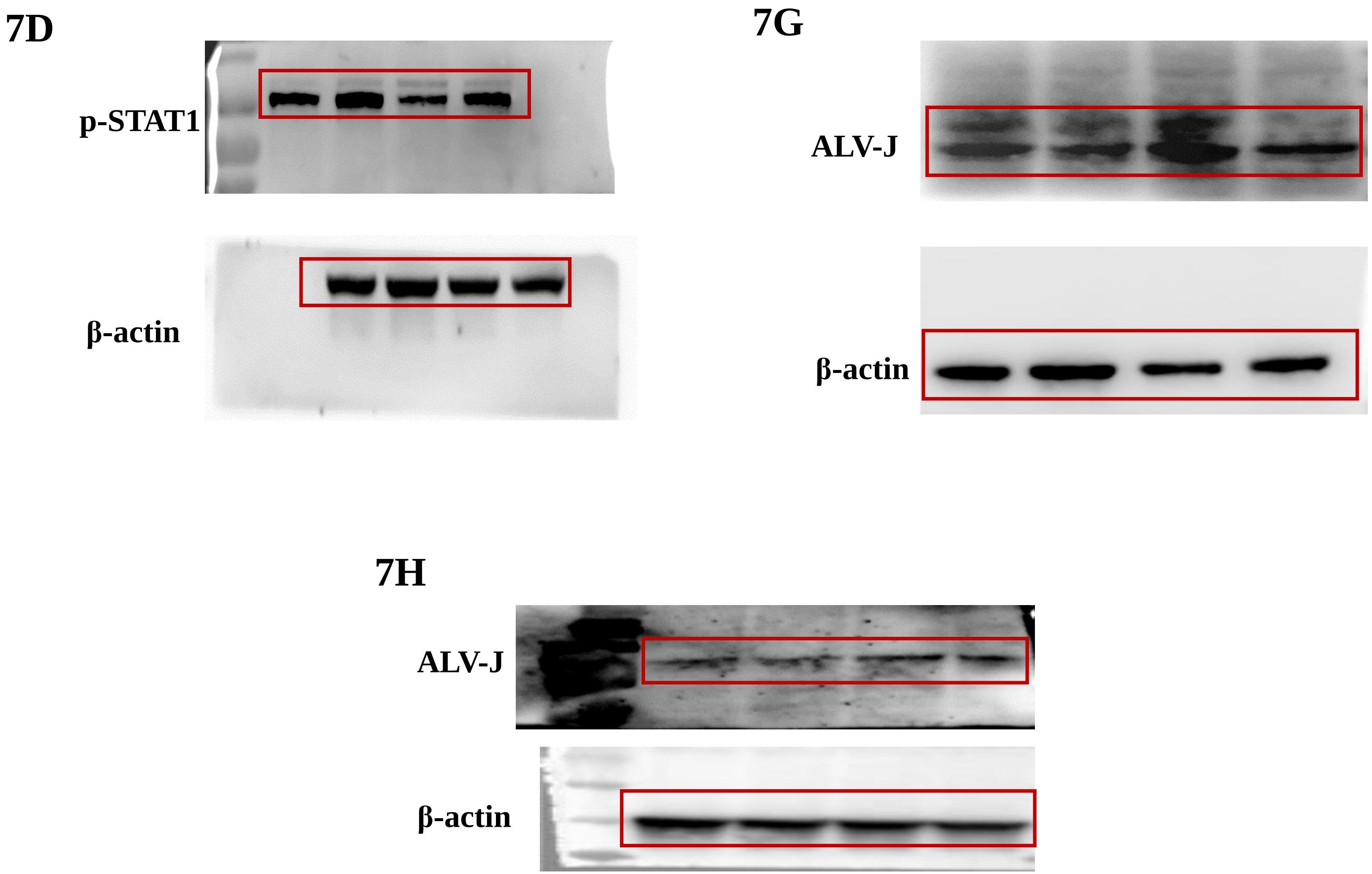

S1 Figure

S1A

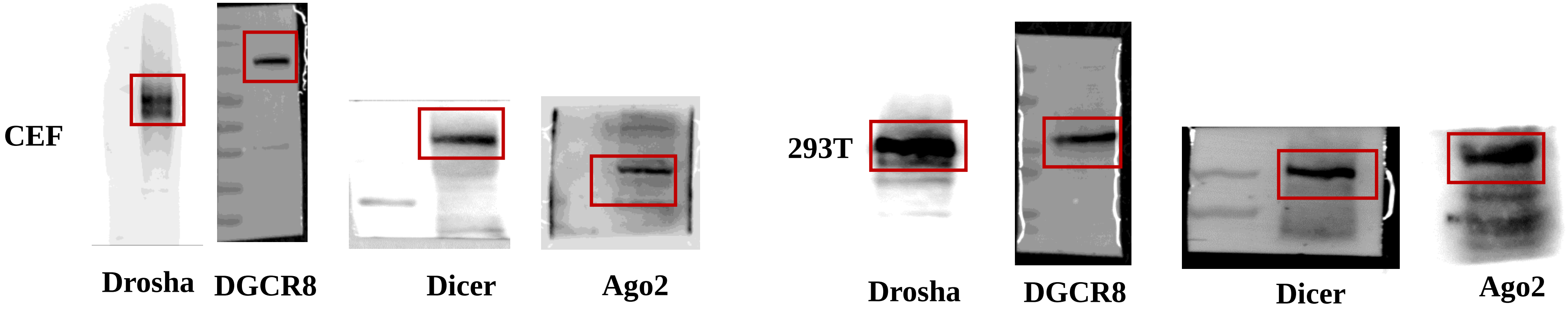

S1B

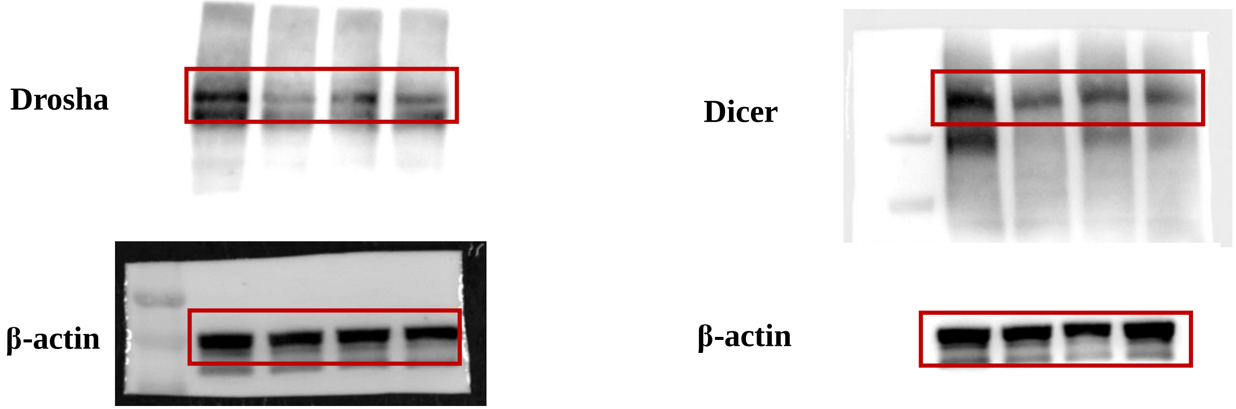

S3 Figure

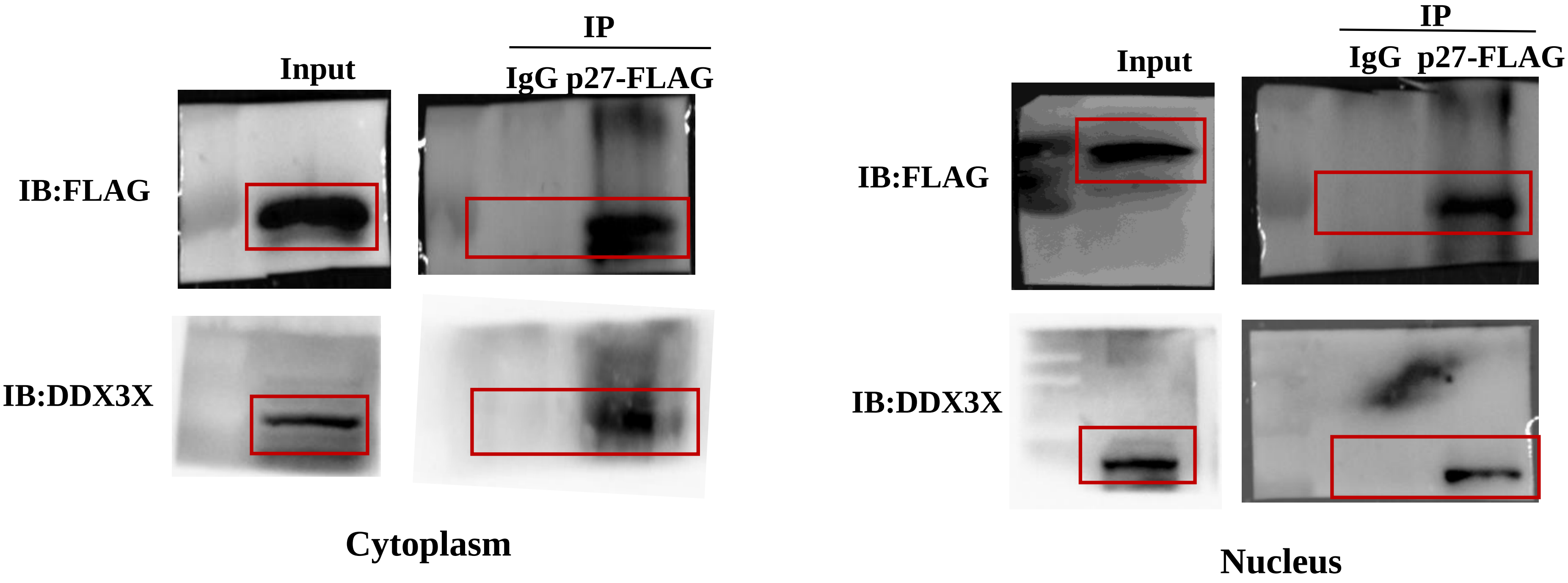

IP
Input
IgG p27-FLAG
IB:FLAG
IB:DDX3X
Cytoplasm
IP
Input
IgG p27-FLAG
IB:FLAG
IB:DDX3X
Nucleus

S4 Figure

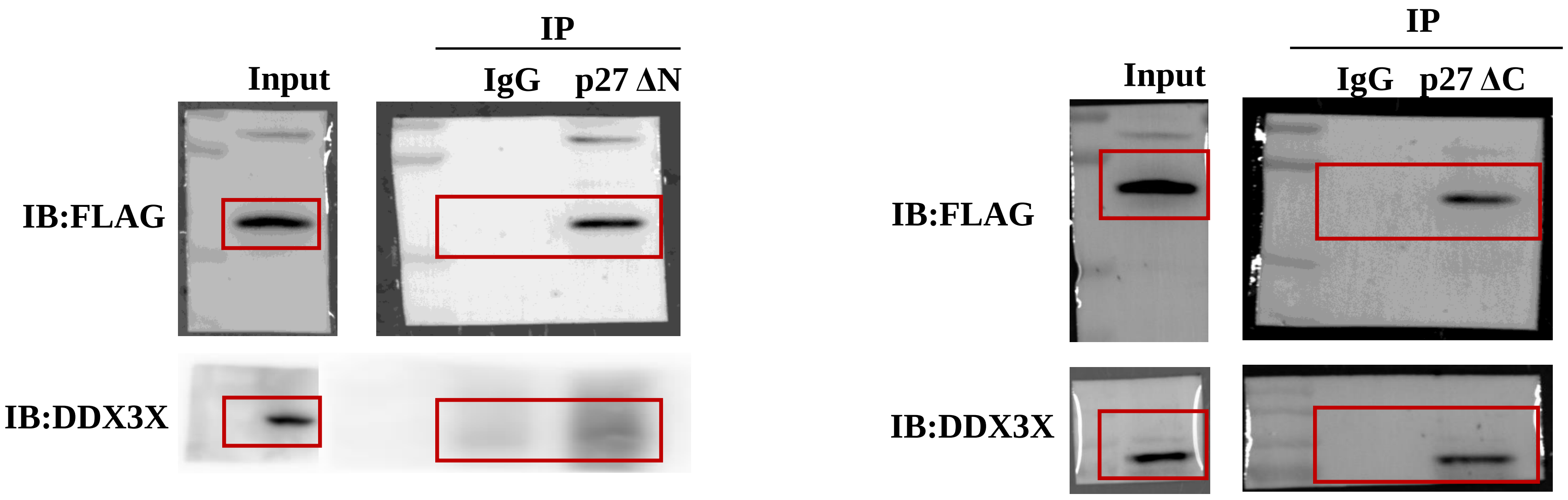
IP
Input
IgG
p27 ΔN
IB:FLAG
IB:DDX3X
IP
Input
IgG
p27 ΔC
IB:FLAG
IB:DDX3X

S5 Figure

S5B

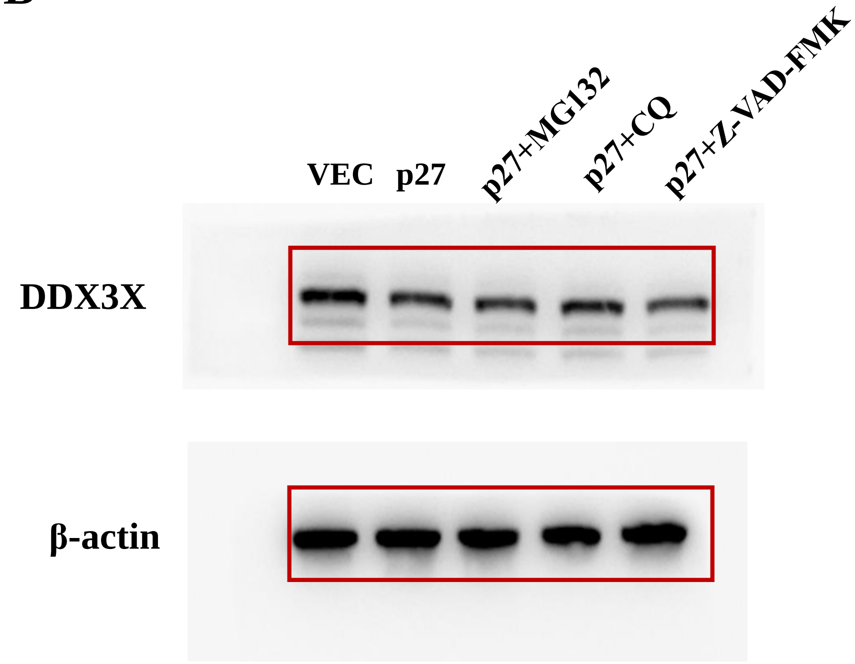

S6 Figure

S6A

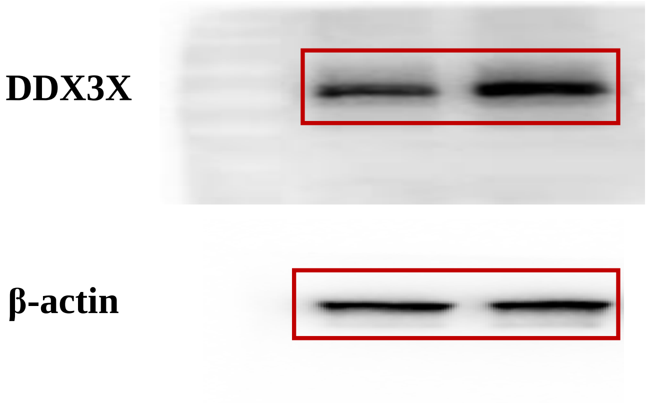

S6B

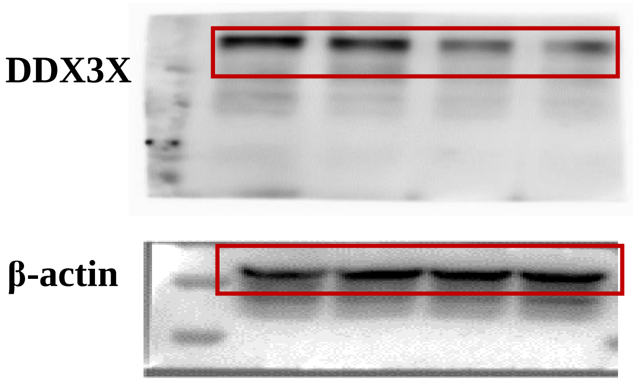

S9 Figure

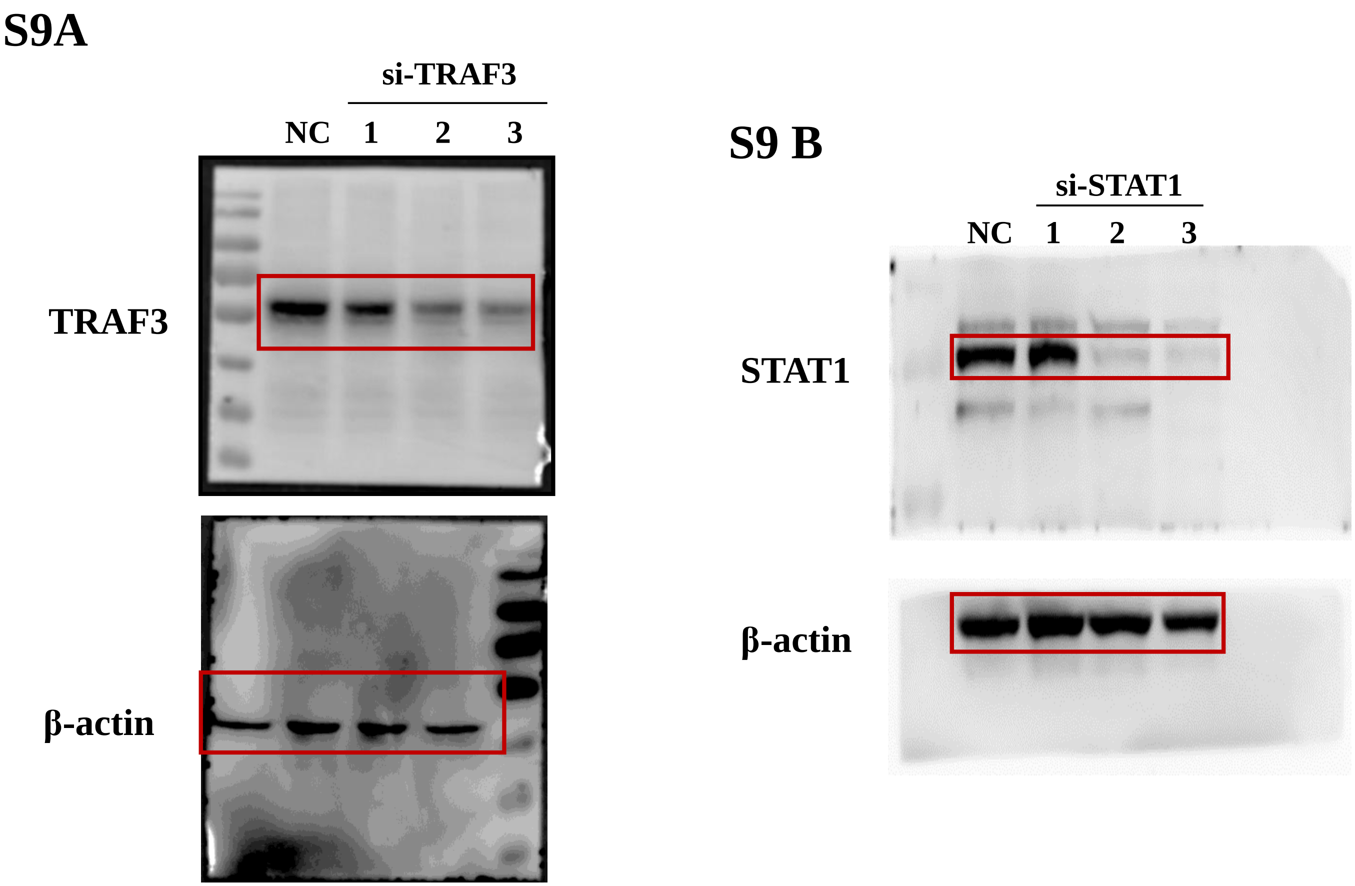
S9A
si-TRAF3
NC 1 2 3
TRAF3
β-actin
S9 B
si-STAT1
NC 1 2 3
STAT1
β-actin

S10 Figure

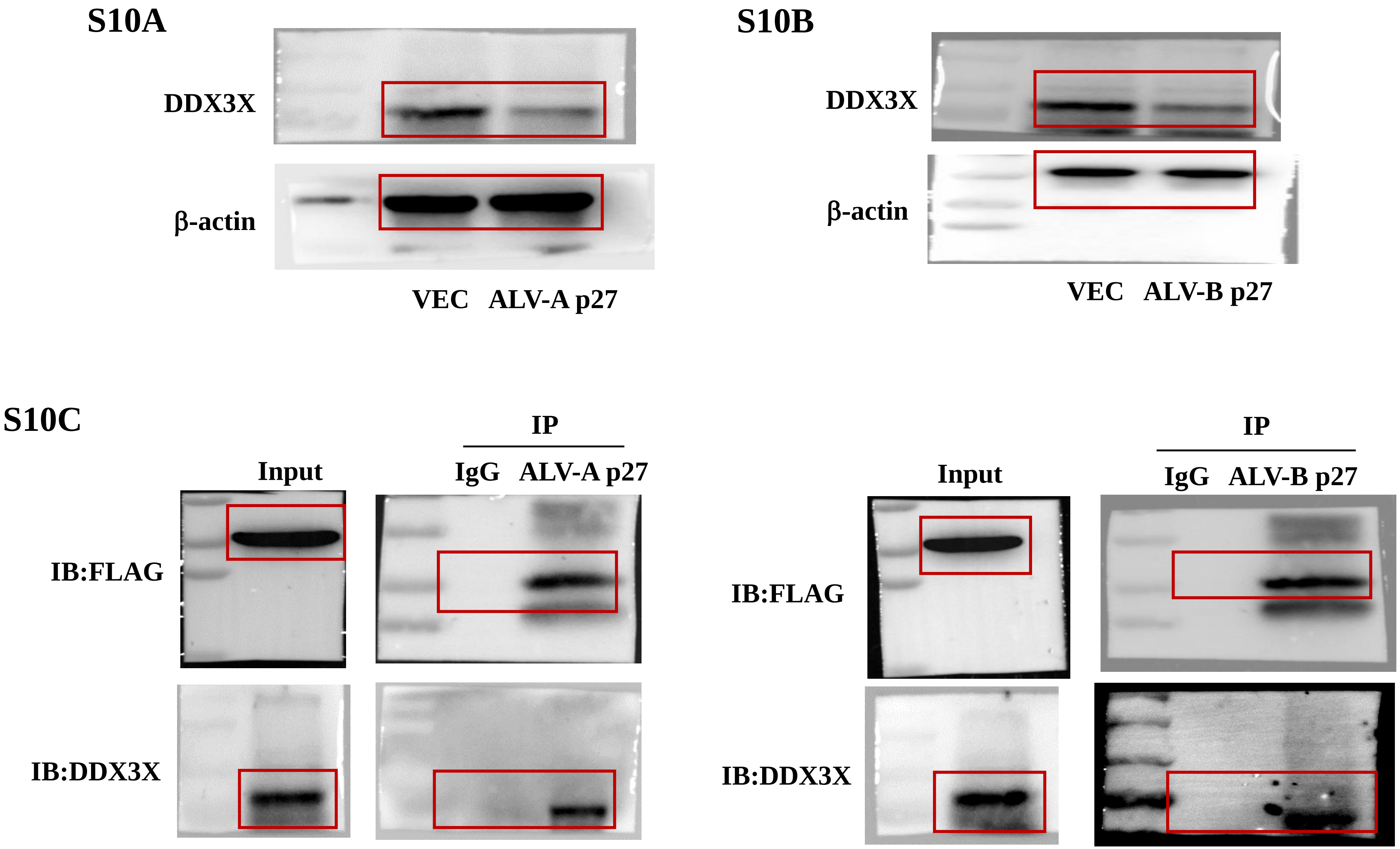
S10A
DDX3X
β-actin
VEC
ALV-A p27
S10B
DDX3X
β-actin
VEC
ALV-B p27
S10C
Input
IP
IgG
ALV-A p27
IB:FLAG
IB:DDX3X
Input
IP
IgG
ALV-B p27
IB:FLAG
IB:DDX3X
